# Role of the Transforming Growth Factor-β in regulating hepatocellular carcinoma oxidative metabolism

**DOI:** 10.1038/s41598-017-12837-y

**Published:** 2017-10-02

**Authors:** Jitka Soukupova, Andrea Malfettone, Petra Hyroššová, María-Isabel Hernández-Alvarez, Irene Peñuelas-Haro, Esther Bertran, Alexandra Junza, Jordi Capellades, Gianluigi Giannelli, Oscar Yanes, Antonio Zorzano, José Carlos Perales, Isabel Fabregat

**Affiliations:** 1 Bellvitge Biomedical Research Institute (IDIBELL), L’Hospitalet, Barcelona, Spain; 2 Department of Physiological Sciences, School of Medicine and Health Sciences, University of Barcelona, Barcelona, Spain; 3 Institute for Research in Biomedicine (IRB Barcelona), The Barcelona Institute of Science and Technology, Barcelona, Spain; 4 Metabolomics Platform, Department of Electronic Engineering (DEEEA), Universitat Rovira i Virgili, Tarragona, Spain; 5 Biomedical Research Centre in Diabetes and Associated Metabolic Disorders (CIBERDEM), Madrid, Spain; 6 National Institute of Gastroenterology IRCCS “S. De Bellis”, Castellana Grotte Bari, Italy; 7 Departament de Bioquímica i Biomedicina Molecular, Facultat de Biologia, Universitat de Barcelona, Barcelona, Spain

## Abstract

Transforming Growth Factor beta (TGF-β) induces tumor cell migration and invasion. However, its role in inducing metabolic reprogramming is poorly understood. Here we analyzed the metabolic profile of hepatocellular carcinoma (HCC) cells that show differences in TGF-β expression. Oxygen consumption rate (OCR), extracellular acidification rate (ECAR), metabolomics and transcriptomics were performed. Results indicated that the switch from an epithelial to a mesenchymal/migratory phenotype in HCC cells is characterized by reduced mitochondrial respiration, without significant differences in glycolytic activity. Concomitantly, enhanced glutamine anaplerosis and biosynthetic use of TCA metabolites were proved through analysis of metabolite levels, as well as metabolic fluxes from U-13C6-Glucose and U-13C5-Glutamine. This correlated with increase in glutaminase 1 (*GLS1*) expression, whose inhibition reduced cell migration. Experiments where TGF-β function was activated with extracellular TGF-β1 or inhibited through TGF-β receptor I silencing showed that TGF-β induces a switch from oxidative metabolism, coincident with a decrease in OCR and the upregulation of glutamine transporter Solute Carrier Family 7 Member 5 (*SLC7A5*) and *GLS1*. TGF-β also regulated the expression of key genes involved in the flux of glycolytic intermediates and fatty acid metabolism. Together, these results indicate that autocrine activation of the TGF-β pathway regulates oxidative metabolism in HCC cells.

## Introduction

Transforming Growth Factor beta (TGF-β) plays a dual role in liver tumorigenesis^1^, since it inhibits growth and induces apoptosis in early stages of carcinogenesis, but once cells acquire the capacity to overcome its suppressor effects, they respond to it undergoing epithelial-mesenchymal transition (EMT), which increases their migratory and invasive potential. For these reasons, targeting TGF-β has recently emerged as a promising tool in fighting liver cancer^2^. In spite of its well known effects on cell proliferation, migration and invasion, little is known about its capacity to induce metabolic reprogramming. Cancer cells modulate their metabolism to favor growth, survival, proliferation and long-term maintenance. The common feature of this alteration is increased glucose uptake and fermentation of glucose to lactate, as well as the use of glycolysis and tricarboxylic acid (TCA) cycle intermediates for biosynthesis and ATP production^3,4^.

Mortality owing to liver cancer has increased in the past years, and the latest estimates indicate that the global health burden of this disease will continue to grow^5^. Hepatocellular carcinoma (HCC) is the most frequent liver tumor. It is generally diagnosed in advanced stages of the disease and, with the exception of sorafenib, no success has been obtained with targeted therapeutic drugs^5^. Likely reasons for this include inter-tumour heterogeneity, as well as a lack of predictive biomarkers of response. Recent evidences indicate metabolic reprogramming as a transcriptional hallmark of HCC^6,7^, which may help to identify the most aggressive lesions in early phases of hepatic carcinogenesis^8^. The principal metabolic alterations in HCC tumors would include elevated glycolysis and reduced TCA cycle flux^9^. However, the main executers of this metabolic reprogramming are not yet known. Proteomic analysis of a subgroup of HCC patients that presented activation of the β-catenin pathway identified glucose metabolism as a key metabolic pathway altered in these patients^10^. A previous study had indicated that activation of the TGF-β pathway may concur in a subgroup of patients that show alterations of the β-catenin/Wnt pathway^11^. However, there is no data at present on the potential role of TGF-β in regulating glycolytic/TCA pathways in HCC.

Aim of this work was to perform a detailed analysis of the differences in basal metabolism amongst HCC cell lines that present differential expression of TGF-β, correlating with distinct epithelial/mesenchymal and migratory phenotype. The potential role of TGF-β in regulating their metabolic profiles was explored in experiments where TGF-β function was activated with extracellular TGF-β1 or inhibited through targeting-knockdown of the TGF-β receptor I (*TGFβRI*).

## Results

### Aerobic glycolysis and oxidative metabolism in HCC cells that show differences in the expression of TGF-β

**Figure 1:**
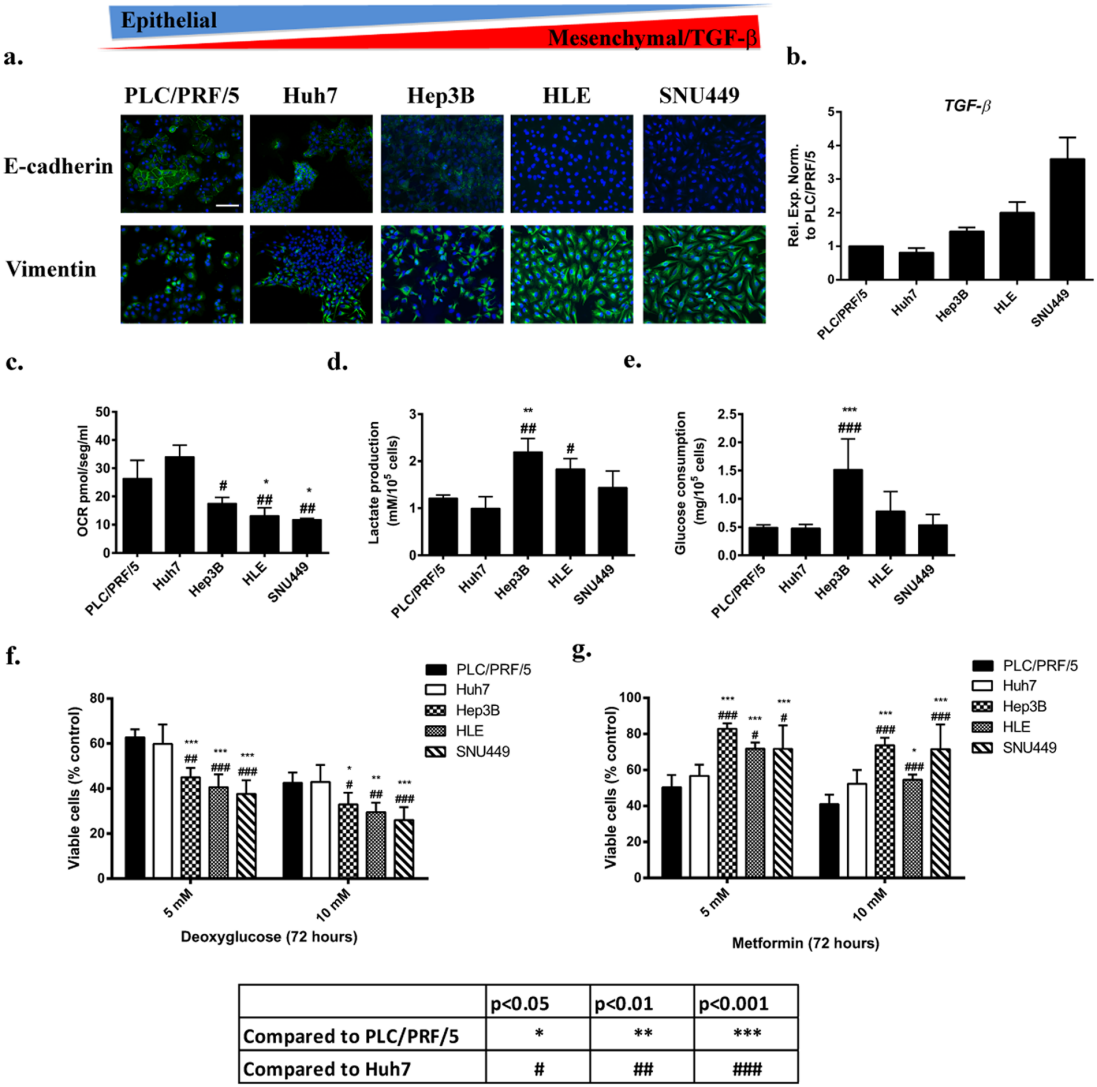
Glycolytic and oxidative metabolism in different HCC cell lines. **(a)** Immunofluorescence analysis of E-cadherin (green) and Vimentin (green). DAPI (blue). Scale bar represents 50 μM. **(b)** mRNA expression levels of TGF-β detected by qRT-PCR and normalized to PLC/PRF/5. Mean ± SD (n = 3). **(c)** OCR was analyzed at basal condition by high resolution respirometry (Oxygraph 2k) in DMEM supplemented with 10% FBS in 5 × 10^5^ cells in suspension. Results are represented as OCR pmol/seg/ml of the first 10 min of respiration after stabilization. **(d,e)** Lactate production and glucose consumption was analyzed after 48 hours of cell culture in DMEM supplemented with 10% FBS and normalized to cell count. **(f)** Cells were treated with 2-DG in a range of concentration (0–10 mM). Cell viability was analyzed after 72 hours by crystal violet staining and normalized to un-treated control. **(g)** Cells were treated with metformin in a range of concentration (0–10 mM). Cell viability was analyzed after 72 hours by crystal violet staining and normalized to un-treated control. Mean ± SD (n = 3, p values are explained in the table below figure).

The metabolic status of representative HCC cell lines that presented differences in their epithelial/mesenchymal phenotype and expression of TGF-β was analyzed (Fig. 1 and Supplementary Fig. S1). Cells expressing higher levels of TGF-β showed a mesenchymal-like morphology, coincident with loss of E-cadherin expression and increased levels of vimentin (Fig. 1). Increased transcript levels of N-cadherin (*CDH2)* and vimentin (*VIM)* and lower levels of E-cadherin (*CDH1)* were observed in the more mesenchymal cells, which correlated with higher expression of the EMT-related transcription factors Snail (*SNAI1*) and Twist1 (*TWIST1*) and differences in their migratory capacity (Supplementary Fig. S1). High resolution respirometry (Oxygraph-2k) revealed that basal oxygen consumption rate (OCR) was significantly higher in epithelial PLC/PRF/5 and Huh7 cells when compared to the other cell lines with a mesenchymal gene expression profile, Hep3B, HLE and SNU449 (Fig. 1c). The amount of lactate released into the cell culture medium after 48 hours was slightly increased in the mesenchymal cells (Fig. 1d), although increased glucose consumption was only observed in the Hep3B cells (Fig. 1e). However, glucose consumption/lactate production ratio was similar in all the cell lines.

Inhibition of glycolysis with 2-deoxy-D-glucose (2-DG, a glucose analog that is not metabolized beyond the initial phosphorylation step) significantly compromised the proliferative capacity of mesenchymal HCC cells to a higher degree than in the epithelial ones (Fig. 1f and Supplementary Fig. S2). Consistently, metformin (a commonly used antidiabetic drug that inhibits mitochondrial Complex I of the respiratory chain) affected more the proliferation of epithelial cell lines characterized by higher oxidative phosphorylation (OXPHOS) activity (Fig. 1g and Supplementary Fig. S3).

### Differences in oxidative metabolism between the epithelial PLC/PRF/5 and the mesenchymal SNU449 cell lines

**Figure 2:**
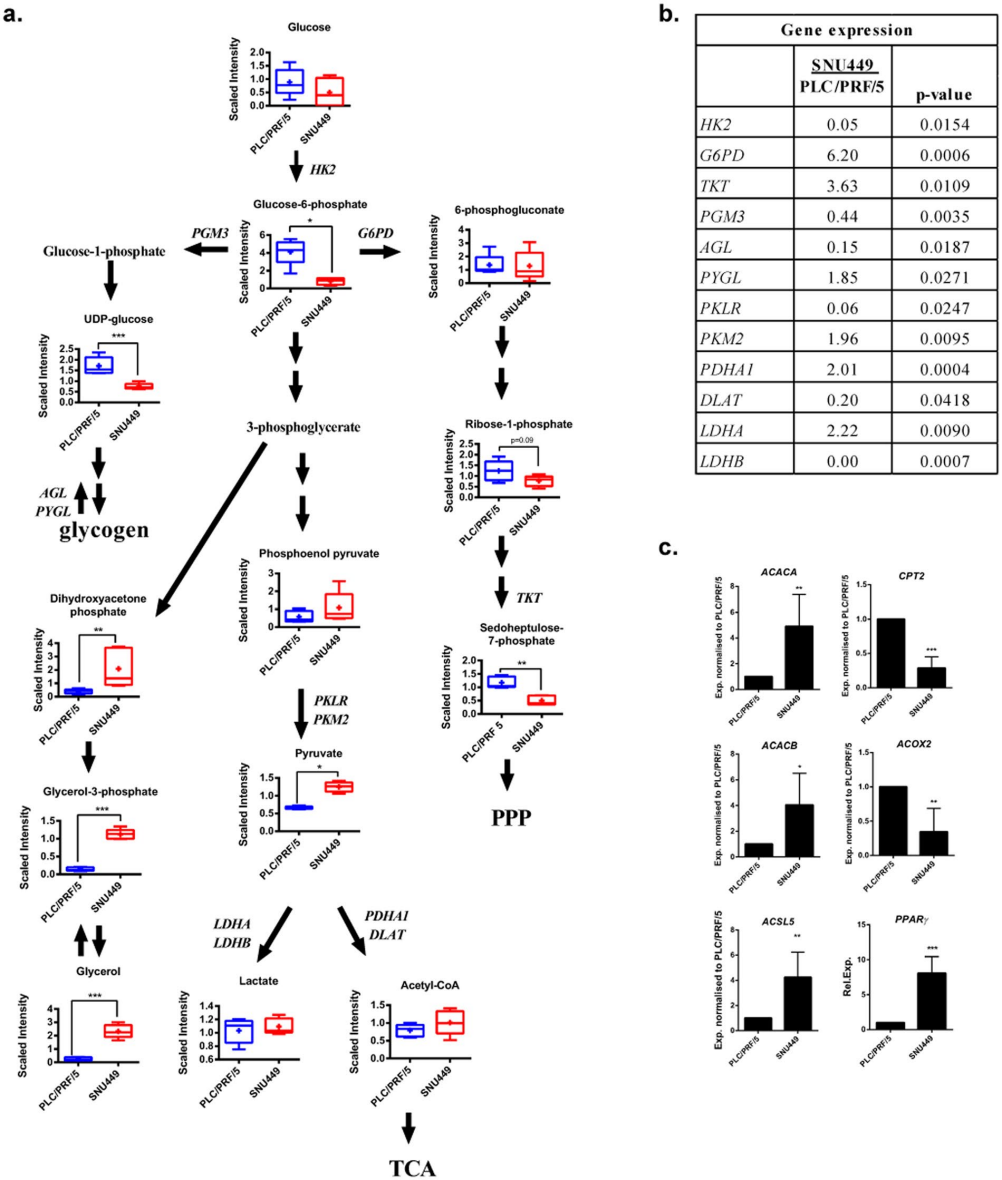
Metabolomic and transcriptomic analysis of PLC/PRF/5 and SNU449 cells: glucose and fatty acid metabolism. **(a)** Schematic diagram of selected metabolites of the glycolytic pathway presented as in the KEGG database (http://www.genome.jp/kegg/). The level of metabolites is depicted by a box plot with whiskers (min to max). Welch’s two-sample *t*-test was used to identify biochemicals that differed significantly between experimental groups (n = 5 for each group). *p < 0.05, **p < 0.01, ***p < 0.001. **(b)** Expression of selected genes related to the glycolytic pathway. Values < 1 indicate lower expression and values > 1 indicate higher expression, SNU449 as compared to PLC/PRF/5. (n = 3, p value indicated in the right column). **(c)** Expression of selected genes related to fatty acid β-oxidation and fatty acid synthesis was detected by qRT-PCR and normalized to PLC/PRF/5. Mean ± SD. (n at least 3). *p < 0.05, **p < 0.01, ***p < 0.001.

To gain insight into the mechanisms that could explain the observed differences in oxidative metabolism among the different HCC cell lines, we performed a metabolomic and transcriptomic analysis in the epithelial PLC/PRF/5 and the mesenchymal SNU449 cells. A full list of the metabolites and genes analyzed is presented in Supplementary Tables S1 and S2, respectively. Selected metabolites related to the glycolytic pathway that were significantly different between PLC/PRF/5 and SNU449 cells, together with key genes showing significant changes in their expression levels, are shown in Fig. 2a,b. Glucose-6-phosphate levels were significantly lower in SNU449 cells (Fig. 2a), even though glucose consumption or lactate production rates were unchanged (Fig. 1e). An increased shunt of glucose-6-phosphate to the pentose phosphate pathway (PPP) was observed in these cells, as indicated by an increased expression of glucose-6-phosphate dehydrogenase (*G6PD*) and other PPP genes, such as transketolase (*TKT*) (Fig. 2b). Consistently, lower levels of some PPP intermediates, such as ribose-1-P or sedoheptulose-7-P, were measured in SNU449 cells, suggesting increased flux through this pathway. On the contrary, PLC/PRF/5 cells had higher phosphoglucomutase 3 (*PGM3*) and alpha-1,6-glucosidase (*AGL*) expression, as well as decreased glycogen phosphorylase (*PYGL*), indicative of enhanced conversion of glucose-6-phosphate to glucose-1-phosphate, and storage of glucose in the form of glycogen. Accordingly, UDP-glucose was also significantly increased in PLC/PRF/5 cells.

Increased PPP pathway found in the SNU449 cells did not correlate with differences in proliferation (data not shown), but with a switch from free fatty acid (FFA) oxidation to synthesis. Indeed, expression of enzymes related to β-oxidation of FFA, such as carnitine palmitoyltransferase 2 *(CPT2)* or acyl-CoA oxidase 2 (*ACOX2*) was lower, whereas expression of genes coding enzymes involved in FFA synthesis, such as acetyl-CoA carboxylase alpha (*ACACA)*, acetyl-CoA carboxylase beta (*ACACB)* and acyl-CoA synthetase long-chain 5 (*ACSL5)*, were higher in SNU449 than in PLC/PRF/5 cells (Fig. 2c). According to this expression profile, SNU449 presented significant higher mRNA levels of the adipogenic transcription factor peroxisome proliferator-activated receptor gamma (PPARγ). In fact, FFA levels were much higher in SNU449 cells than in PLC/PRF/5 cells, which also presented significantly higher amounts of several phospholipids and sphingolipids (Supplementary Fig. S4). Additionally, SNU449 cells presented larger metabolite pools in the glycerol synthesis pathway, such as dihydroxyacetone phosphate, glycerol-3-phosphate and glycerol, necessary for glycerolipid synthesis (Fig. 2a). Together, these results suggest that the increased PPP in mesenchymal SNU449 cells may be necessary to supply NADPH for lipid synthesis.

Significant differences in the expression of pyruvate kinase isoenzymes were also observed. Liver isoform of pyruvate kinase (*PKLR*) was expressed in the epithelial PLC/PRF/5 cells, but was almost absent in the SNU449, which predominantly expressed pyruvate kinase muscle 2 (*PKM2*) isotype (Fig. 2b and Supplementary Fig. S5). Even though pyruvate dehydrogenase A1 (*PDHA1*) expression was lower in PLC/PRF/5 cells, the expression of the component of the pyruvate dehydrogenase complex, dihydrolipoamide S-Acetyltransferase (*DLAT*), was increased in PLC/PRF/5 cells. Differential expression of lactate dehydrogenase isoenzymes was also noted: epithelial PLC/PRF/5 expressed lactate dehydrogenase B (*LDHB*), which was absent in SNU449 (Fig. 2b and Supplementary Fig. S5). However, despite these observations, no significant differences were observed in intracellular pyruvate or lactate levels.

**Figure 3:**
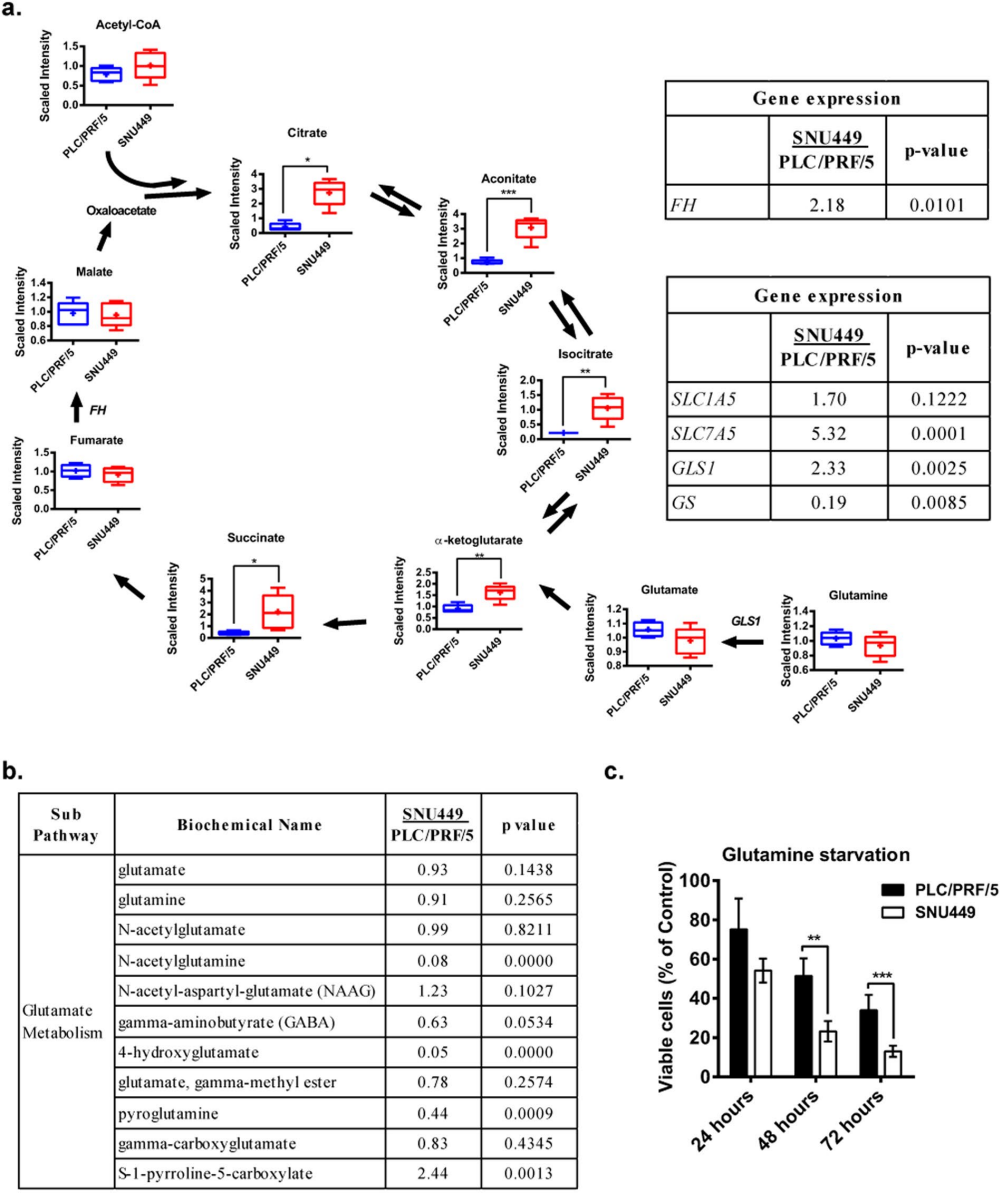
Metabolomic and transcriptomic analysis of PLC/PRF/5 and SNU449 cells: differences in the TCA cycle and glutamine metabolism. **(a**) Left: Schematic diagram of the TCA cycle as presented in the KEGG database (http://www.genome.jp/kegg/). The level of metabolites is depicted by a box plot with whiskers (min to max). Welch’s two-sample *t*-test was used to identify metabolites that differed significantly between experimental groups (n = 5 for each group). *p < 0.05, **p < 0.01, ***p < 0.001. Right: Expression of *FH* and selected genes related to the glutamine metabolism pathway. Values < 1 indicate lower expression and values > 1 indicate higher expression, SNU449 as compared to PLC/PRF/5. (n = 3, p value indicated in the right column). **(b)** Metabolites from the glutamine/glutamate pathway presented in fold comparing SNU449 to PLC/PRF/5. Welch’s two-sample *t*-test was used to identify metabolites that differed significantly between experimental groups (n = 5 for each group, p value indicated in the right column). **(c)** PLC/PRF/5 cells and SNU449 were cultivated in DMEM medium (25 mM glucose) without glutamine supplemented with 10% FBS. Cell viability was analyzed after 24, 48 and 72 hours by crystal violet staining and normalized to control (2 mM glutamine). Mean ± SD (n = 3), **p < 0.01, ***p < 0.001.

SNU449 cells showed increased levels of citrate, aconitate, isocitrate, α-ketoglutarate and succinate, TCA cycle intermediates (Fig. 3a), without relevant changes in the expression of the genes coding for the enzymes involved in this cycle. This would suggest increased TCA carbon anaplerosis in SNU449 cells. Indeed, the expression of glutaminase 1 (*GLS1*) that converts glutamine to glutamate was higher in SNU449 cells (Fig. 3a), suggesting that glutamine anaplerosis might be responsible for increased TCA intermediates pool size. Analysis of the expression of glutamine synthase (GS), responsible for production of endogenous glutamine, revealed that SNU449 cells showed significantly lower mRNA levels than PLC/PRF/5 cells (Fig. 3a), suggesting that SNU449 might be more dependent on exogenous glutamine due to the lack of GS for endogenous glutamine production, In contrast, the expression of the amino acid transporter Solute Carrier Family 7 Member 5 (*SLC7A5*), a member of the Sodium-Independent Neutral Amino Acid Transporter *LAT 1* complex that exchanges glutamine by other essential amino acids (EAA), was increased in SNU449. These results indicate that the main source of glutamine for anaplerosis in SNU449 cells is extracellular. Metabolites from the glutamine metabolism pathway, such as N-acetylglutamine, 4-hydroxyglutamate and pyroglutamine, were found to be decreased in SNU449 cells (Fig. 3b). Accordingly, these cells presented significantly reduced cell viability after 48 and 72 hours of glutamine depletion compared to PLC/PRF/5 (Fig. 3c). Furthermore, glutamine anaplerosis favors the migratory phenotype of the SNU449 cells, since treating them with a selective inhibitor of GLS1 (BPTES, 10 μM, 12 hours treatment, which at this time it does not induce changes in cell viability), significantly decreased cell migration, as assessed by the real time migration assay (xCELLigence System) (Supplementary Fig. S6).

**Figure 4:**
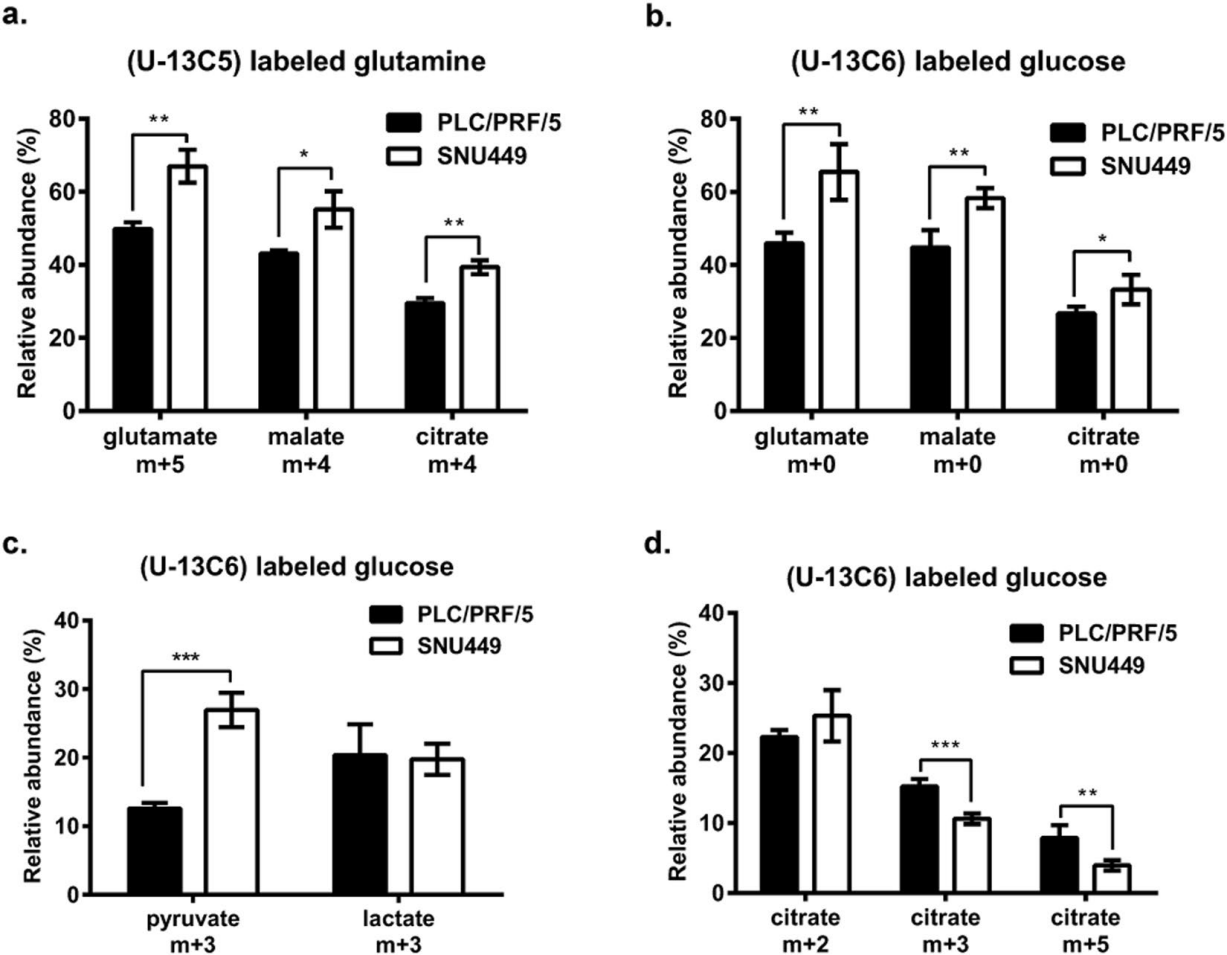
13C isotopomer distribution from fully labeled glutamine/glucose. **(a)** PLC/PRF/5 and SNU449 cells were exposed to 4 mM fully labeled glutamine (U-13C5-Glutamine) in a medium containing 25 mM glucose and 10% dialyzed FBS. **(b**–**d**) PLC/PRF/5 and SNU449 cells were exposed to 25 mM fully labeled glucose (U-13C6-Glucose) in a medium containing 4 mM glutamine and 10% dialyzed FBS. As ^13^C from glutamine/glucose is distributed among various metabolites, their mass increases proportionally to the number of incorporated carbons. This increase in mass was detected by GC-MS. Isotope distribution in metabolites is marked as m + x, where the m stands for natural mass of the metabolite and the x indicates number of incorporated ^13^C carbons. Mean ± SD. *p < 0.05, **p < 0.01, ***p < 0.001.

To further elucidate the dynamics of the TCA cycle and glutamine anaplerosis in PLC/PRF/5 versus SNU449 cells, we performed isotopologue enrichment analysis using universally labeled glutamine (U-13C5-Glutamine) and glucose (U-13C6-Glucose). Stable isotope labeling of key intermediates of the TCA cycle and glycolysis were quantified using mass spectrometry (MS). As shown in Fig. 4a, isotope enrichment analysis of glutamate, malate and citrate provides strong evidence for increased anaplerosis of glutamine in SNU449 cells. Fully labeled glutamate (m + 5) and malate (m + 4) distribution significantly increased in SNU449 cells, suggesting increased labeled glutamine flux into the TCA cycle. This was further confirmed by increased enrichment in citrate species (m + 4) corresponding to the direct oxaloacetate flux from glutamine. Consistently, when cells were labeled with U-13C6-Glucose, unlabeled species (m + 0) were significantly higher in SNU449 cells, indicating lower contribution of glucose carbons into the TCA cycle intermediate pool (Fig. 4b). Metabolic flux analysis using U-13C6-Glucose confirmed reduced pyruvate enrichment (m + 3), while maintaining identical glucose to lactate flux in PLC/PRF/5 versus SNU449 (Fig. 4c). Further analysis of the contribution of the pyruvate carboxylase (PC) and PDH fluxes into citrate (m + 3 and m + 5 versus m + 2, respectively) demonstrated that PLC/PRF/5 cells have a higher PC directed entry of pyruvate into the TCA cycle (higher enrichment in m + 3 and m + 5 citrate species), whereas PDH flux was unaffected (Fig. 4c,d), suggesting that the accumulation of fully labeled pyruvate in SNU449 was due to reduced entry into the TCA cycle, probably as glutamine anaplerosis was substantially higher. TCA cycling, identified by the distribution of m + 2 species (2^nd^ turn of the cycle), was not different between both cell lines (data not shown), suggesting that increased glutamine carbon flux in SNU449 was directed towards biosynthetic processes.

In summary, these results indicate that epithelial and mesenchymal HCC cell lines differ in their glucose and glutamine utilization pathways, correlating with differential expression of key regulatory genes. Results also indicate that the TCA cycle supports a more biosynthetic role in the mesenchymal cell line, coincident with increased glutamine anaplerosis, which would be essential for the cell migratory phenotype.

### Role of TGF-β on the metabolic adaptations of HCC cells

We next wondered whether the observed differences in metabolism between these epithelial/mesenchymal cell lines might be attributed to the TGF-β produced by those cells (see Fig. 1b). Therefore, we chronically treated an epithelial PLC/PRF/5 cell line with TGF-β (2 ng/ml) for two weeks (gain of function). TGF-β treated PLC/PRF/5 (TβT-PLC) cells presented up-regulation of characteristic mesenchymal genes, such as *CDH2, VIM* or *TWIST1*, although the expression of *CDH1* was maintained (Supplementary Fig. S7a,b). Importantly, in spite of decreased cell proliferation, due to the inhibitory effects of TGF-β, TβT-PLC cells showed enhanced migratory capacity (Supplementary Fig. S7c,d). In parallel, *TGFβRI* was stably downregulated in the mesenchymal HCC cell line, SNU449 (loss of function: SNU449sh-control cells transfected with unspecific shRNA; SNU449shTβRI- silenced cells transfected with specific shRNA). *TGFβRI* silencing did not provoke a full mesenchymal-epithelial transition (MET), although we could observe decreased expression of key EMT-related transcription factors, such as *SNAI1* and *TWIST1* and the reorganization of F-actin in pericellular area. Furthermore, SNU449shTβRI cells presented significantly decreased cell migration capacity, with no changes in cell proliferation (Supplementary Fig. S8).

**Figure 5:**
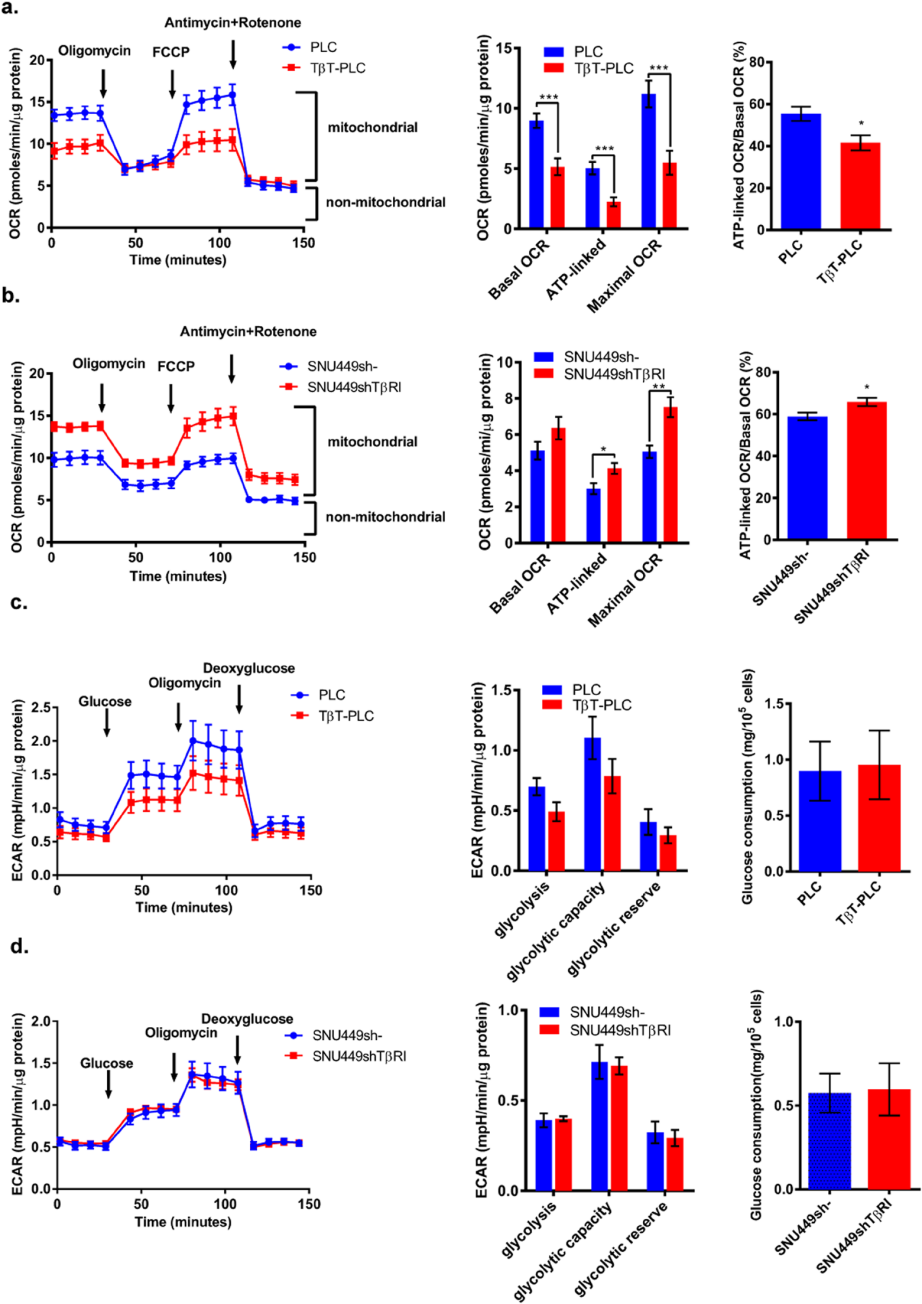
Seahorse analysis of OXPHOS and glycolysis in HCC cells. Role of the TGF-β pathway. **(a,b)** OCR normalized to protein content in PLC, TβT-PLC **(a)** and SNU449sh-, SNU449shTβRI cells **(b)** incubated 30 minutes prior experiment in XF assay medium supplemented with 5 mM glucose and 2 mM glutamine and consecutively injected with oligomycin (1 μM), FCCP (1.5 μM), antimycin (1 μM) and rotenone (1 μM). Continuous OCR values (pmoles/min/µg protein) are shown. Mitochondrial functions were analysed as explained in Supplementary materials and methods. The % of ATP-linked OCR was calculated as ATP-linked OCR/basal OCR. Mean ± SEM (n at least 6 from three independent experiments). *p < 0.05, **p < 0.01, ***p < 0.001. **(c,d)** ECAR in PLC, TβT-PLC **(c)** and SNU449sh-, SNU449shTβRI cells **(d)** incubated 30 minutes prior experiment in XF assay medium supplemented with 2 mM glutamine and consecutively injected with glucose (10 mM), oligomycin (1 μM) and deoxyglucose (50 mM). Mean ± SEM (n at least 9 from three independent experiments). Continuous ECAR values (mpH/min/µg protein) are shown. Glycolytic functions were analysed as explained in Supplementary materials and methods. Mean ± SEM (n = 9 from three independent experiments). Glucose consumption (mg/10^5^ cells) was measured after 48 hours of culture in DMEM supplemented with 10% FBS and normalized to cell number. Mean ± SD (n = 3).

To better explore mitochondrial physiology, we measured oxygen consumption rates (OCR) during sequential treatment with compounds that modulate mitochondrial activity using a Seahorse apparatus (more details in the Methods section). PLC/PRF/5 cells showed high basal mitochondrial oxygen consumption that was 50% coupled to ATP production. Chronic activation of these cells with TGF-β induced a significant decrease in basal, ATP-linked and maximal OCR (FCCP treated) (Fig. 5a). Control PLC cells were able to increase OCR after FCCP treatment (maximal OCR), indicating spare respiratory capacity. However, TβT-PLC cells did not show a significant increase in OCR after FCCP addition. Mesenchymal SNU449 cells showed lower basal OCR (similar in value to the one observed in TβT-PLC cells) and they were not able to increase OCR after addition of FCCP. Knockdown of *TGFβRI* in SNU449 cells led to a significant increase in ATP-linked and maximal OCR, without affecting basal OCR (Fig. 5b). No differences were observed in basal extracellular acidification rate (ECAR) or lactate production at 48 hours among the cell lines (Supplementary Fig. S9), suggesting that differences in mitochondrial respiration do not correlate with opposing changes in glycolytic capacity. This was confirmed when we measured ECAR during sequential treatment with compounds that modulate glycolytic activity (Fig. 5c,d). In TβT-PLC cells we observed a tendency to lower ECAR, and no changes were observed in glycolytic capacity or in glycolytic reserve. In SNU449shTβRI cells, no differences in ECAR were observed when compared to SNU449sh- control cells.

Altogether, these results indicate that the TGF-β pathway regulates mitochondrial respiratory capacity in HCC cells.

### Metabolomics and transcriptomics analyses suggest a role for TGF-β in regulating key metabolic pathways in HCC cells

Metabolomic analysis of PLC versus TβT-PLC and SNU449sh- versus SNU449shTβRI cells revealed a number of metabolites significantly altered (Supplementary Fig. S10a). Pathway set enrichment analysis displayed the number of experimentally regulated compounds relative to all detected compounds in a pathway (Supplementary Fig. S10b). The unsupervised hierarchical clustering was performed using Euclidean distance and showing differences between the respective groups (Supplementary Fig. S10c).

**Figure 6:**
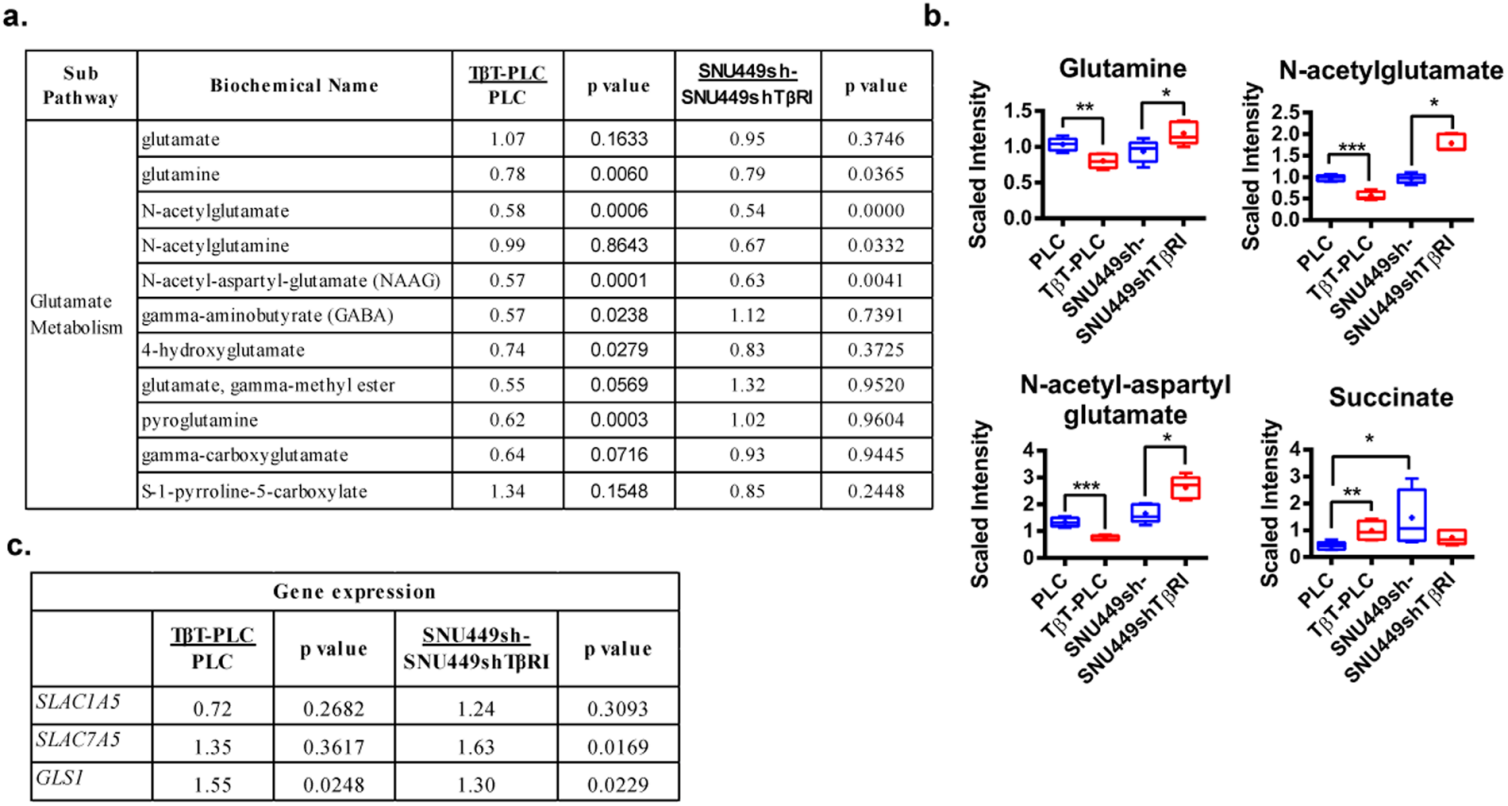
Role of the TGF-β pathway on glutamine metabolism in HCC cells. TβT-PLC cells were compared to PLC cells. SNU449sh- cells were compared to SNU449shTβRI cells. **(a)** Metabolites from the glutamine/glutamate metabolism pathway presented in fold. Welch’s two-sample *t*-test was used to identify metabolites that differed significantly between experimental groups (n = 5 for each group, p value indicated in the right column). **(b)** The level of respective metabolites that changed significantly in both conditions is depicted by a box plot with whiskers (min to max). Mean ± SD (n = 5 for each group); *p < 0.05, **p < 0.01, ***p < 0.001. **(c)** Expression of selected genes related to the glutamine metabolism. Values < 1 indicates lower expression and values > 1 indicates higher expression, as compared to respective controls (n = 3, p value indicated in the right column).

We used a p-value of 0.05 as a cut-off to analyze consistent changes induced by TGF-β (with increased levels in TβT-PLC cells and decreased levels in SNU449shTβRI cells and vice versa: Supplementary Table S3). Interestingly, levels of amino acids were generally decreased in TGF-β exposed cells (TβT-PLC versus PLC and SNU449sh- versus SNU449shTβRI), namely threonine, N-acetylalanine, N-acetylasparagine, glutamine, N-acetylglutamine, N-acetyl-aspartyl-glutamate (NAAG), N6, N6, N6-trimethyllysine and methionine sulfone. Importantly, the observed changes in downstream glutamate metabolites (Fig. 6a,b) were accompanied by changes in gene expression (Fig. 6c). *GLS1* expression was increased in TβT-PLC cells and decreased in SNU449shTβRI cells. Additionally, SNU449shTβRI showed reduced expression of glutamine transporter *SLC7A5*. These results strongly suggest a role for TGF-β in regulating glutamine metabolism in HCC cells. It is worth noting that activation of the TGF-β pathway (TβT-PLC and SNU449sh-) correlated with significantly higher levels of succinate, suggesting an increased entry of glutamine to the TCA cycle (Fig. 6b).

**Figure 7:**
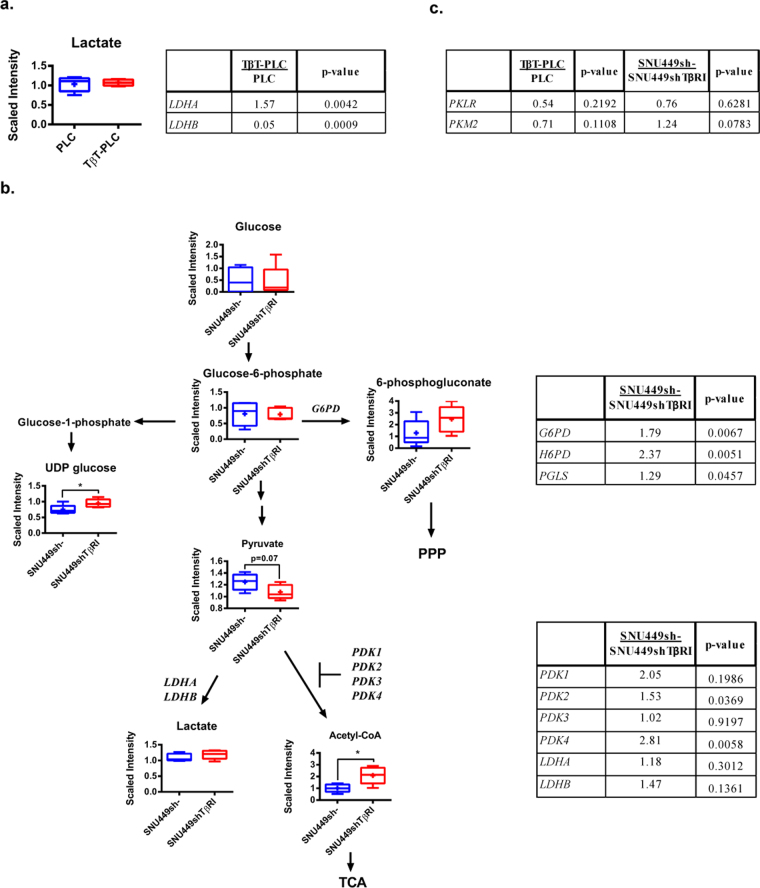
Role of TGF-β in the regulation of glucose metabolism. (**a**) Left: Lactate levels between PLC and TβT-PLC are depicted by a box plot with whiskers (min to max). Welch’s two-sample *t*-test was used to identify biochemicals that differed significantly between experimental groups (n = 5 for each group). Right: Expression of *LDHA* and *LDHB*. Values < 1 indicate lower expression and values > 1 indicate higher expression, TβT-PLC versus PLC (n = 3, p value indicated in the right column). (**b**) Left: Schematic diagram of selected metabolites of the glycolytic pathway presented as in the KEGG database (http://www.genome.jp/kegg/). The level of respective metabolites between SNU449sh- and SNU449shTβRI is depicted by a box plot with whiskers (min to max). Welch’s two-sample *t*-test was used to identify biochemicals that differed significantly between experimental groups (n = 5 for each group). *p < 0.05 Right: Expression of selected genes related to the specific parts of the pathway. Values < 1 indicate lower expression and values > 1 indicate higher expression, SNU449sh- versus SNU449shTβRI (n = 3, p value indicated in the right column). **(c)** Expression of *PKLR* and *PKM2* in TβT-PLC versus PLC and in SNU449sh- versus SNU449shTβRI. Values < 1 indicate lower expression and values > 1 indicate higher expression. (n = 3, p value indicated in the right column).

Moreover, additional changes in glucose metabolism might be under the control of TGF-β. The most interesting finding after chronic activation of PLC/PRF/5 cells with TGF-β was the switch in the expression of *LDHA* and *LDHB* (Fig. 7a), reminiscent of the expression pattern observed in SNU449 cells (Fig. 2), although no differences in lactate levels were detected. Inhibition of the TGF-β pathway in SNU449shTβRI cells correlated with reduced expression of PPP-related genes, such as *G6PD*, hexose-6-phosphate dehydrogenase (*H6PD)* and 6-phosphogluconolactonase (*PGLS)*, which indicated a role for the autocrine TGF-β pathway in pushing glucose through the PPP. Furthermore, in SNU449shTβRI cells, decreased levels of sphingolipids and phospholipids were detected (Supplementary Fig. S11 a + b) together with decreased expression of genes that play a regulatory role in FFA synthesis, such as *ACSL5* and *PPARγ* (Supplementary Fig. S11c). These results together indicate that the autocrine TGF-β pathway in SNU449 cells regulates NADPH synthesis, through an increase of the oxidative PPP pathway, and lipid synthesis.

Pyruvate levels presented a tendency to decrease (86% of control, p = 0.07), which could be due to an increase in pyruvate to acetylCoA conversion. Pyruvate dehydrogenase kinase 2 (*PDK2)* and pyruvate dehydrogenase kinase 4 (*PDK4)* were significantly decreased, resulting in increased pyruvate dehydrogenase activity. Indeed, the level of acetylCoA was increased in the SNU449shTβRI cells (Fig. 7b). Interestingly, UDP-glucose levels significantly increased after *TGFβRI* silencing.

All together, these results indicate that autocrine activation of the TGF-β pathway may be responsible for many of the differences observed in the pattern of metabolites and gene expression found in SNU449 when compared to the PLC/PRF/5 cells (Fig. 2). However, in the case of PKs, although a trend towards an increase in the expression of *PKLR* and a decrease in *PKM2* expression was observed in the SNU449shTβRI, changes were not significant and the expression of these enzymes did not change in the TβT-PLC cells (Fig. 7c). Indeed, changes in the PKs enzyme expression were not under the control of the TGF-β pathway.

## Discussion

Tumor cells adapt their metabolic pathways to support oncogenic growth under adverse situations and to take full advantage of their energy reserves in new functions, such as cell migration and invasion^3,4^. HCC is a highly heterogeneous cancer, represented by strong differences among the patients both in phenotype and in molecular profiles^5^. In previous studies from our group we postulated that differences in the expression of TGF-β correlate with differences in the expression of mesenchymal genes both in HCC cell lines and in HCC patients^12^. Furthermore, autocrine expression of TGF-β is responsible for differences in the stem and migratory phenotype of HCC cells^13,14^. Although several clues suggest that metabolic reprogramming may be a transcriptional hallmark in the process to liver carcinogenesis^6–8,15^, there is still no explanation for the heterogeneity of metabolic profiles found among the different HCC phenotypes, nor the metabolic adaptations of HCC cells to support their migration and invasion.

Our study focuses on this issue, exploring the differences of oxidative metabolism among different HCC cell lines that present differential epithelial/mesenchymal characteristics, as well as different autocrine production of TGF-β. Results indicate that the switch from an epithelial to a mesenchymal/migratory phenotype in HCC cells is characterized by reduced mitochondrial respiration and enhanced glutamine anaplerosis, proved through analysis of metabolite levels, as well as metabolic fluxes from U-13C6-Glucose and U-13C5-Glutamine, which suggest an enhanced biosynthetic use of TCA metabolites. Interestingly, these changes occur in the absence of significant differences in glycolytic activity. Glycolysis was similar in a panel of HCC cells and metabolic flux analysis using U-13C6-Glucose revealed identical glucose to lactate flux in the epithelial PLC/PRF/5 versus the mesenchymal SNU449. Pyruvate carboxylase flux would explain the increased OCR in PLC/PRF/5 cells.

Gain of TGF-β function experiments demonstrate that when an epithelial (low TGF-β production) cell line is chronically incubated with TGF-β, significant decreases in OCR are observed at all levels: basal, ATP-linked and maximal. By contrary, targeting knock-down the *TGFβRI* in a mesenchymal cell line provokes an increase in maximal OCR, as well as in the percentage of OCR dedicated to ATP synthesis. In both cases, glycolytic activity does not suffer relevant changes. It is noteworthy that both gain and loss of function experiments consistently reflect a role for TGF-β in regulating glutamine metabolism. Indeed, differences observed between epithelial and mesenchymal cells on glutamate/glutamine metabolites are mimicked by TGF-β treatment of the epithelial cells and are reversed by silencing *TGFβRI* in the mesenchymal cells. The main target of TGF-β to mediate these changes could be *GLS1* and the glutamine transporter *SLC7A5*. Glutamine is the major systemic carrier of nitrogen. It is also a key nutrient for numerous intracellular processes, including oxidative metabolism and ATP generation, but also biosynthesis of proteins, lipids and nucleic acids^16^. The demand of this amino acid can become so great during tumorigenesis that many cancer cells are glutamine addicted^16^. Glutaminolysis starts with the conversion of glutamine to glutamate, catalyzed by the glutaminases (*GLS1* and *GLS2*) in the mitochondria. Previous studies have shown that GLSs are expressed in a wide variety of tumors, correlating with tumor growth, however its regulation in cancer cells is not well understood^17^. Worth mentioning that none of the HCC cells used in this study express *GLS2*, which would be an advantage to the tumor cells, since it has been recently proposed that *GLS2* exhibits a tumor suppressive function^18^. In fact, a glutaminolysis independent function of *GLS2* inhibits migration and invasion of HCC cells by repressing EMT^19^. A metabolic switch from *GLS2* to *GLS1* was observed in a series of tissues representing progressive pathologic states mimicking HCC oncogenic transformation; moreover high expression of *GLS1* and low expression of *GLS2* in HCC was found to correlate with survival in HCC patients^20^. Here we show that TGF-β upregulates *GLS1* in liver tumor cells. Through increasing glutamine transport and *GLS1* expression, TGF-β would be favoring increased glutamate content, which would push carbons through the TCA cycle. In agreement with this, succinate levels are increased under all the conditions where the TGF-β pathway is activated. Since the ATP-linked OCR decreases in cells where the TGF-β pathway is more active, TCA may support other purposes, probably biosynthetic pathways. This metabolic switch would offer advantages to the migratory cells, as confirmed by our results revealing a significant decrease in the migratory capacity of SNU449 cells when GLS1 activity is inhibited, similar to the decrease observed when the expression of the *TGFβRI* is attenuated with shRNA. It has been recently proposed that mitochondrial catabolic capacity may be a limiting constraint preventing complete substrate catabolism in high uptake metabolism in cancer cells, whereas glutamine addiction may provide resistance to metabolic stress through excess redox and energy production^21^.

A comparison of the metabolite and gene expression profile between the more epithelial and the more mesenchymal HCC cells reflects clear differences in the utilization of glycolytic metabolites. Indeed, epithelial cells, such as PLC/PRF/5, are using glucose to store it in glycogen, whereas mesenchymal/migratory cells, such as SNU449, direct it towards the PPP cycle, correlating with higher glycerol metabolism and lipid biosynthesis. *G6PD* and *TKT* transcript levels, involved in the PPP cycle, are significantly higher in the mesenchymal/migratory cells when compared to the epithelial ones. Furthermore, genes related to FFA oxidation show lower expression, concomitant with increased expression of some genes related to FFA biosynthesis. All these data suggest that the mesenchymal/migratory phenotype would require higher lipogenesis. In this line of evidence, it has been recently suggested that treatment of human liver cancer cell lines with FFAs exacerbate the EMT phenotype, whereas chemical inhibition of CD36 mitigated these effects^22^. In human HCC samples, increased *G6PD* expression correlates with grading, metastasis and poor prognosis^8^ and decreased *G6PD* expression results in inhibition of hepatoma cell migration and invasion^23^. Our results indicate that regulation of the *G6PD* and *H6PD* expression is dependent on TGF-β, since attenuation of the pathway with *TGFβR1* shRNA significantly decreases their transcript levels. Furthermore, storage of glycogen might be increased after *TGFβRI* silencing, as reflected by the increase in UDP-glucose levels. Finally, TGF-β also regulates genes related to lipid synthesis, such as *ACSL5* and *PPARγ*, and altogether contributes to the observed decrease in lipid content in SNU449 cells after *TGFβRI* silencing.

Another relevant finding of our study is the role that TGF-β would play in switching the expression of the LDH genes, favoring *LDHA* expression. Lactate dehydrogenases catalyze the inter-conversion of pyruvate to lactate and NADH to NAD^+^. Different tissues in the body have different metabolic rates, energy requirements and functions, which are often reflected by the LDHA/LDHB ratio. LDHA is known as the M subunit and, although predominantly found in the heart, it plays many roles both in non-neoplastic and cancer cells^24^. *LDHA* is elevated in many types of cancers and has been linked to tumor growth, maintenance, and invasion; therefore, its inhibition may restrict the energy supply in tumors and thereby reduce the metastatic and invasive potential of cancer cells^25^. Supporting this idea, knockdown of *LDHA* induces apoptosis in HCC cells and suppresses tumor growth and metastasis in a xenograft mouse model^26^. Furthermore, low expression of *LDHB* correlates with unfavorable survival in HCC^27^. Little is known on the factors that regulate the increase in *LDHA* and/or decrease in *LDHB* expression in HCC and here we propose that TGF-β would be one of these factors. There are multiple evidences that support this hypothesis: i) *LDHA* transcript levels are higher in mesenchymal HCC cells, which barely express *LDHB*, correlating with TGF-β expression; ii) a significant switch in the *LDHA/LDHB* ratio is observed after chronic stimulation of HCC cells with TGF-β. Silencing TGFβRI in mesenchymal HCC cells induces a modest, not statistically significant, increase in *LDHB* expression without changing *LDHA* transcript levels, which would indicate that in advanced stages other factors may contribute to maintain the expression of this gene. Further study is required to better understand the relevance of this *LDHA/LDHB* switch in HCC cells, since our result indicate that it does not correlate with significant differences in lactate levels.

Our study also identifies some differences in the metabolic profile between epithelial and mesenchymal/migratory HCC cells that would not be related to differential expression of TGF-β in these cells. *PKLR* expression was absent in the mesenchymal SNU449 cells, which predominantly expressed *PKM2*, which is the more abundant form of PK in cancer cells, conferring substantial benefits on the rapidly growing tumorigenic cells. In contrast, forced expression of the *PKM1* isoform has been associated with reduced tumor cell proliferation^28^. It has been recently suggested that switching of pyruvate kinase isoform L to M2 promotes metabolic reprogramming in hepatocarcinogenesis^29^. Furthermore, *PKM2* expression indicates poor prognosis in HCC and would mediate metastasis^30^. Our results do not support a clear role for TGF-β in up-regulating *PKM2* expression. Indeed, extracellular TGF-β does not produce changes in pyruvate levels and does not increase *PKM2* expression in the epithelial cell line. Furthermore, although silencing *TGFβRI* in the mesenchymal cell line produces a decrease in the pyruvate and in *PKM2* transcript levels, changes are not statistically significant.

In summary, our study clearly supports that many of the differences observed in the oxidative metabolism among epithelial and mesenchymal HCC cells may be explained by differences in the degree of activation of the TGF-β pathway. We propose a new protumorigenic role for TGF-β in HCC cells: through decreasing mitochondrial respiration and enhancing glutamine anaplerosis and PPP cycle, TGF-β switches cell metabolism to a more biosynthetic profile, which would favor the migratory and invasive phenotype.

## Materials and Methods

### Cell culture

PLC/PRF/5, Hep3B and SNU449 cells were obtained from the European Collection of Cell Cultures (ECACC). Huh7 and HLE cells were from the Japanese Collection of Research Bioresources Cell Bank (JCRB Cell Bank). Cell lines were never used in the laboratory for longer than four months after receipt or resuscitation. Characteristics of all the cell lines are presented in Supplementary Table S5. All cells were maintained in DMEM media (Lonza, Basel, Switzerland) supplemented with 10% FBS (Sera Laboratories International Ltd, West Sussex, UK), Penicillin (100 U/ml), Streptomycin (100 μg/ml) and Amphotericin (2.5 μg/ml) and L-glutamine (2 mM). They were maintained in a humidified atmosphere at 37 °C, 5% CO_2_. For chronical TGF-β treatment, human recombinant TGF–β1 (Calbiochem, La Jolla, USA) was used at 2 ng/ml in a medium and replaced every 48 hours. Cells were observed under an Olympus 70iX microscope. Methods for immunofluorescence, western blot analysis and flow cytometry analysis are included in Supplementary materials and methods.

### Targeted know-down assays

For stable transfection of short hairpin RNA (shRNA), cells at 50–60% confluence were transfected with MAtra-A reagent (IBA GmbH, Goettingen, Germany) at a dilution of 1:600 in complete media, according to the manufacturer’s recommendation, using 2 µg/ml of shRNA plasmid, as previously described^12^. Additional information in Supplementary materials and methods.

### Analysis of gene expression

A total RNA was extracted using E.Z.N.A Total RNA kit I (OMEGA Biotek, Nocross, USA) according to the manufacturer’s protocol. Reverse transcription (RT) was done using the High Capacity Reverse Transcriptase kit (Applied Biosystems, Foster City, USA), and 1 μg of total RNA from each sample for complementary DNA synthesis. For qRT-PCR, expression levels were determined in duplicate in a LightCycler 480 Real-time PCR system, using the LightCycler 480 SYBR Green I Master (Roche, Basel, Switzerland) and normalized to housekeeping gene *L32*. The list of primers used in the study in Supplementary materials and methods. The expression of glutamine synthase (*GS*) was analyzed using a TaqMan gene expression assay (Hs00365928_g1) according to manufacturer´s instructions.

### RT profiler array

A total RNA was extracted using E.Z.N.A Total RNA kit I (OMEGA Biotek, Nocross, USA) according to the manufacturer’s protocol and reverse transcribed to cDNA using Reverse transcriptase First strand kit (Qiagen, Hilden, Germany). The human glucose metabolism RT^2^ Profiler PCR Array (Qiagen, Hilden, Germany) was used to screen 84 genes in a LightCycler 480 Real-time PCR system using the LightCycler 480 SYBR Green I Master (Roche, Basel, Switzerland). For data analysis, fold-changes in each gene expression were calculated using the ΔΔCt method, after normalization to housekeeping gene (*RPLP0*) using a data analysis RT^2^ profiler platform (http://pcrdataanalysis.sabiosciences.com/pcr/arrayanalysis.php).

### Migration assay

Real-time assay of cell motility was examined through the xCELLigence System (ACEA Biosciences, San Diego, USA) as previously described^12^. 4 × 10^4^ cells/well were seeded onto the top chamber of a CIM plate®, which was coated with a collagen IV solution (Sigma-Aldrich) and placed onto the Real-Time Cell Analyzer (RTCA) station (ACEA Biosciences, San Diego, USA). Continuous values were represented as Cell Index (CI), a dimensionless parameter, which reflects a relative change in measured electrical impedance, and quantified as a slope (h^−1^) of the first 8 hours of cell migration.

### Metabolic profiling

Cell samples (100 μl of cell pellet, five replicates for each group) were submitted for metabolic profiling to Metabolon Inc. In brief, samples were extracted in methanol and the resulting extract was divided into five fractions: two for analysis by two separate reverse phase (RP)/UPLC-MS/MS methods with positive ion mode electrospray ionization (ESI), one for analysis by RP/UPLC-MS/MS with negative ion mode ESI, one for analysis by HILIC/UPLC-MS/MS with negative ion mode ESI, and one sample was reserved for backup. Proprietary software was used to match ions to an in-house library of standards for metabolite identification and quantification. Following normalization to Bradford protein concentration, log transformation and imputation of missing values, if any, with the minimum observed value for each compound, a Welch´s two-sample-t-test was used to identify molecules that differed significantly between experimental groups.

### Metabolic flux assay

Cells have been seeded in 10 cm Petri dish. When confluent, cells were washed 3 times with PBS and treatment medium was added. For glutamine labeling DMEM medium with 25 mM glucose containing 10% dialyzed FBS and 4 mM fully labeled glutamine (Cambridge Isotope Laboratories Inc., product reference CLM-1822-H-PK) was used. For glucose labeling DMEM medium without glucose containing 10% dialyzed FBS, 4 mM glutamine and 25 mM fully labeled glucose (Cambridge Isotope Laboratories Inc., product reference CML-1396-PK) was used. Cells were incubated for 6 hours at 37 °C and 5% CO_2_. After 6 hours, cells were trypsinized and washed 3 times with cold PBS. Cell pellets were frozen and stored at −80 °C until metabolites extraction.

Metabolites were extracted by adding 300 µl of cold methanol/water (8:1, v:v). Samples were vortexed for 30 seconds and immersed in liquid N_2_ to disrupt cell membranes followed by 10 seconds of bath-sonication. These two steps were repeated 3 times. Cell lysates were incubated for 20 minutes in ice before centrifugation (5000 g, 15 minutes at 4 °C). 10 µl of ^13^C-glycerol (150 ppm) was added to the supernatant as internal standard. Next, 250 µl of each sample were dried under a stream of N_2_ gas. Lyophilized polar extracts were incubated with 50 µL methoxyamine in pyridine (40 µg/µl) for 45 minutes at 60 °C. To increase volatility of the compounds, we silylated the samples using 25 µl N-methyl-N-trimethylsilyltrifluoroacetamide with 1% trimethylchlorosilane (Thermo Fisher Scientific) for 30 minutes at 60 °C. A 7890 A GC system coupled to a 7000 QqQ mass spectrometer (Agilent Technologies, Palo Alto, CA.) was used for isotopologue determination. Derivatized samples were injected (1 µl) in the gas chromatograph system with a split inlet equipped with a J&W Scientific DB5−MS+DG stationary phase column (30 mm × 0.25 mm i.d., 0.1 µm film, Agilent Technologies). Helium was used as a carrier gas at a flow rate of 1 ml/minute in constant flow mode. The injector split ratio was adjusted to 1:5 and oven temperature was programmed at 70 °C for 1 minute and increased at 10 °C/minute to 325 °C. The ionization performed was positive chemical ionization (CI) with isobutene as reagent gas. Mass spectral data on the 7000 QqQ were acquired in scan mode monitoring selected ion clusters of the different metabolites.

### High-resolution respirometry

To determine OCR, 5 × 10^5^ cells were resuspended in 1 ml fresh warm medium (DMEM supplemented with 10% FBS and 2 mM glutamine) and placed into Oxygraph chamber (Oxygraph-2k, Oroboros, Innsbruck, Austria) pre-equilibrated with 21% oxygen, equipped with a thermostat control (stable 37 °C), a micro stirring device and a oxygen electrode disk. After stabilization of the instrument, cells were left to respire for 10 minutes to monitor the oxygen consumption. The experiment was repeated at least three times.

### Seahorse analysis

The Seahorse analyzer XF24 (Agilent, Santa Clara, USA) was used to continuously monitor OCR and ECAR. Two days prior to the experiment, 20 000 cells/ well were seeded in a XF24 cell culture plate in DMEM media supplemented with 10% FBS, 25 mM glucose, 2 mM glutamine and cultivated at 37 °C in humidified atmosphere and 5% CO_2_. One day prior to the experiment, 1 ml of XF calibrator was added to each well of the XF cartridge and incubated overnight at 37 °C in humidified atmosphere and 0% CO_2_. 30 minutes prior to the experiment, cells were washed with PBS and 625 μl of respective XF assay medium was added per well and incubated for 30 minutes at 37 °C in humidified atmosphere and 0% CO_2_. For the XF Cell Mito Stress analysis, XF assay medium was supplemented with 5 mM glucose and 2 mM glutamine. For XF Glycolysis stress kit XF assay medium was supplemented with 2 mM glutamine. After 15 min equilibration time, OCR and ECAR were accessed every 8:30 minutes (after 3 minutes mixing, 2 minutes wait, 3:30 minutes measure), always 4 times after the addition of the respective compounds. The different compounds were added to the injection ports of the XF cartridge in 10x of final concentration and were diluted prior the experiment in XF assay medium. After the Seahorse experiment, all the cells were recovered, firstly the medium with any floating cells was centrifuged, and remaining cells were lysed using a lysis buffer (0.1 N NaOH, 0.1% SDS). Protein concentration was determined using Pierce BCA protein assay kit (Thermo Fisher Scientific) for normalization. Additional information in Supplementary materials and methods.

### Cell proliferation assay

Cells were seeded in a 24-well plate (10 000 cells/well) in 500 μl of DMEM (Lonza BE12-604) supplemented with 10% FBS, 2 mM glutamine and penicillin/streptomycin (Gibco). After 24 hours, the media was replaced with media containing respective compounds. At specific time points (indicated in figure legends), cells were washed with PBS and stained with crystal violet (0.2% crystal violet in 2% ethanol solution) for 30 minutes and later washed with distilled water and dissolved in 10% SDS on a shaker for 30 min. The absorbance was analyzed on a plate reader at 560 nm.

### Inhibitors used

2-DG (Sigma Aldrich. D8375, 2 M stock solution in distilled water, working concentration 0–10 mM). Metformin (1,1 dimethylbiguanide hydrochloride, Sigma Aldrich D150959). 1 M stock solution in distilled water, working concentration 0–10 mM). BPTES (Bis-2-(5-phenylacetamido-1,3,4-thiadiazol-2-yl)ethyl sulfide, Sigma Aldrich SML0601, stock solution in DMSO, working concentration 10 μM).

### Glutamine deprivation

Cell were seeded in a 24-well plate (10–15 000 cells/well) in 500 μl of full DMEM supplemented with 10% FBS, 2 mM glutamine and penicillin/streptomycin (Gibco). After 24 hours, the cells were washed twice with glutamine-free DMEM (Lonza, 12–614) and incubated for up to 72 hours in glutamine-free medium with penicillin/streptomycin and 10% FBS. As a control, glutamine was added to the glutamine-free DMEM (2 mM).

### Lactate production and glucose consumption assays

Cells were seeded in full media in a 6-well plate (8 × 10^4^ cells in 2 ml) in triplicates. Media was replaced after 24 hours. After 48 hours the media was collected for analysis of L-lactate and glucose and cells were counted for normalization. The concentration of L-lactate was determined using an enzymatic reaction based on the oxidation of L-lactate to pyruvate as previously described^31^. The concentration of glucose was determined using a glucose oxidase and peroxidase method, PGO Enzymes (Sigma Aldrich) according to manufacturer’s instructions. Additional information in Supplementary materials and methods.

### Statistical analyses

All data represent at least three experiments and are expressed as the mean ± standard deviation (SD). Differences between groups were compared using either Student’s t-test or one-way ANOVA associated with the Dunnett’s test. Statistical calculation was performed using GraphPad Prism software (GraphPad Software Inc., La Jolla, USA) and statistical significance was assumed when p < 0.05. Metabolomic profiling results were analyzed by Metabolon Inc. using a Welch’s two-sample-t-test.

## Electronic supplementary material

Supplementary Information

## References

[1] Fabregat I (2016). TGF-β signalling and liver disease. FEBS J..

[2] Giannelli G (2016). The rationale for targeting TGF-β in chronic liver diseases. Eur J Clin Invest.

[3] Pavlova N, Thomson C (2016). The emerging hallmarks of cancer metabolism. Cell Metab.

[4] Liberti M, Locasale J (2016). The Warburg Effect: How Does it Benefit Cancer Cells?. Trends Biochem Sci.

[5] Llovet, J., Villanueva, A., Lachenmayer, A. & Finn, R. Advances in targeted therapies for hepatocellular carcinoma in the genomic era. *Nat Rev Clin Oncol* **12****(****7****)** (2015). 10.1038/nrclinonc.2015.121 26099984

[6] Beyoğlu D, Idle J (2013). The metabolic window into hepatobiliary disease. J Hepatol.

[7] Allain C, Angenard G, Clément B, Coulouarn C (2016). Integrative Genomic Analysis Identifies the Core Transcriptional Hallmarks of Human Hepatocellular Carcinoma. Cancer Res.

[8] Kowalik, M. *et al*. Metabolic reprogramming identifies the most aggressive lesions at early phases of hepatic carcinogenesis. *Oncotarget* **7****(****22****)** (2016). 10.18632/oncotarget.8632 PMC5078020 27070090

[9] Huang Q (2013). Metabolic characterization of hepatocellular carcinoma using nontargeted tissue metabolomics. Cancer Res.

[10] Chafey P (2009). Proteomic analysis of beta-catenin activation in mouse liver by DIGE analysis identifies glucose metabolism as a new target of the Wnt pathway. Proteomics.

[11] Lachenmayer A (2012). Wnt-pathway activation in two molecular classes of hepatocellular carcinoma and experimental modulation by sorafenib. Clin Cancer Res.

[12] Bertran E (2013). Overactivation of the TGF-β pathway confers a mesenchymal-like phenotype and CXCR4-dependent migratory properties to liver tumor cells. Hepatology.

[13] Fernando J (2015). A mesenchymal-like phenotype and expression of CD44 predict lack of apoptotic response to sorafenib in liver tumor cells. Int J Cancer.

[14] Malfettone A (2017). Transforming growth factor-β-induced plasticity causes a migratory stemness phenotype in hepatocellular carcinoma. Cancer Lett.

[15] Xie Q (2017). Multi-omics analyses reveal metabolic alterations regulated by hepatitis B virus core protein in hepatocellular carcinoma cells. Sci Rep.

[16] Lukey M, Wilson K, Cerione R (2013). Therapeutic strategies impacting cancer cell glutamine metabolism. Future Med Chem.

[17] Jin L, Alesi G, Kang S (2016). Glutaminolysis as a target for cancer therapy. Oncogene.

[18] Zhang C (2016). Glutaminase 2 is a novel negative regulator of small GTPase Rac1 and mediates p53 function in suppressing metastasis. Elife.

[19] Kuo T (2016). Glutaminase 2 stabilizes Dicer to repress Snail and metastasis in hepatocellular carcinoma cells. Cancer Lett.

[20] Yu D (2015). Kidney-type glutaminase (GLS1) is a biomarker for pathologic diagnosis and prognosis of hepatocellular carcinoma. Oncotarget.

[21] Zielinski D (2017). System biology analysis of drivers underlying hallmarks of cancer cell metabolism. Sci Rep.

[22] Nath A, Li I, Roberts L, Chan C (2015). Elevated free fatty acid uptake via CD36 promotes epithelial-mesenchymal transition in hepatocellular carcinoma. Sci Rep.

[23] Hu H (2014). Changes in glucose-6-phosphate dehydrogenase expression results in altered behavior of HBV-associated liver cancer cells. Am J Physiol Gastrointest Liver Physiol..

[24] Valvona C, Fillmore H, Nunn P, Pilkington G (2016). The Regulation and Function of Lactate Dehydrogenase A: Therapeutic Potential in Brain Tumor. Brain Pathol.

[25] Miao P, Sheng S, Sun X, Liu J, Huang G (2013). Lactate dehydrogenase A in cancer: a promising target for diagnosis and therapy. IUBMB Life.

[26] Sheng S (2012). Knockdown of lactate dehydrogenase A suppresses tumor growth and metastasis of human hepatocellular carcinoma. FEBS J..

[27] Chen R, Zhou X, Yu Z, Liu J, Huang G (2015). Low Expression of LDHB Correlates With Unfavorable Survival in Hepatocellular Carcinoma: Strobe-Compliant Article. Medicine (Baltimore).

[28] Dayton T, Jacks T, Heiden MV (2016). PKM2, cancer metabolism, and the road ahead. EMBO Rep..

[29] Wong C (2014). Switching of pyruvate kinase isoform L to M2 promotes metabolic reprogramming in hepatocarcinogenesis. PLoS One.

[30] Liu W (2015). PKM2 promotes metastasis by recruiting myeloid-derived suppressor cells and indicates poor prognosis for hepatocellular carcinoma. Oncotarget.

[31] Álvarez Z, Hyroššová P, Perales J, Alcántara S (2016). Neuronal Progenitor Maintenance Requires Lactate Metabolism and PEPCK-M-Directed Cataplerosis. Cereb Cortex.

