# Supplementary material for: Role of the Transforming Growth Factor-β in regulating hepatocellular carcinoma oxidative metabolism
Source: Sci Rep. 2017 Oct 2;7:12486. doi: 10.1038/s41598-017-12837-y (PMC5624948; doi:10.1038/s41598-017-12837-y)
Supplement: Supplementary file 1 — Supplementary Information [file 41598_2017_12837_MOESM1_ESM.pdf]

Role of the Transforming Growth Factor-β in regulating hepatocellular carcinoma oxidative metabolism

Jitka Soukupova,[1] Andrea Malfettone,[1#] Petra Hyroššová,[1,2#] María-Isabel Hernández-Alvarez,[3,5] Irene Peñuelas-Haro,[1] Esther Bertran,[1] Alexandra Junza,[4,5] Jordi Capellades,[4,5] Gianluigi Giannelli,[6] Oscar Yanes,[4,5] Antonio Zorzano,[3,5,7] José Carlos Perales,[1,2] Isabel Fabregat[1, 2,]

# These authors equally contributed to this work.

From:

[1]*Bellvitge Biomedical Research Institute (IDIBELL), L'Hospitalet, Barcelona, Spain;*
[2]*Department of Physiological Sciences, School of Medicine and Health Sciences, University of Barcelona, Barcelona, Spain;*
[3]*Institute for Research in Biomedicine (IRB Barcelona), The Barcelona Institute of Science and Technology, Barcelona, Spain;*
[4] *Metabolomics Platform, Department of Electronic Engineering (DEEEA), Universitat Rovira i Virgili, Tarragona, Spain;*
[5]*Biomedical Research Centre in Diabetes and Associated Metabolic Disorders (CIBERDEM), Madrid, Spain;*
[6]*National Institute of Gastroenterology IRCCS "S. De Bellis" Castellana Grotte Bari, Italy;*
[7]*Departament de Bioquímica i Biomedicina Molecular, Facultat de Biologia, Universitat de Barcelona, Spain;*

Correspondence and requests for materials should be addressed to:

Isabel Fabregat
IDIBELL
Hospital Duran i Reynals
Gran Via de l'Hospitalet, 199
08908 L'Hospitalet
Barcelona
E-mail: ifabregat@idibell.cat

Supplementary Information

Supplementary materials and methods

Immunofluorescence studies

Fluorescence microscopy studies were performed as described previously [1]. For Ki67 and F-actin, cells were fixed with 4% paraformaldehyde in PBS for 30 minutes at room temperature. For Ki67, cells were permeabilized with Triton X-100 (0.1%) and incubated with a blocking solution (1% BSA; 10% FBS) before addition of the primary antibody (1:100 dilution). For F-actin, cells were incubated with a blocking solution before addition of the primary antibody (1:200 dilution). For Vimentin and E-cadherin, cells were fixed with methanol (100%; -20º C, 2 minutes) prior incubation with blocking solution and the addition of the primary antibodies (1:50). All primary antibodies were diluted in 1% BSA (1 hour; room temperature). After primary antibody incubation, cells were washed three times with PBS, and afterwards samples were incubated with Alexa Fluor 488-conjugated anti-rabbit or Alexa Fluor 488-conjugated anti-mouse for 1 hour at room temperature (1:1000) and mounted in Vectashield mounting medium. A list of used primary and secondary antibodies in Supplementary table S6 online. Cells were visualized in a Nikon eclipse 80i microscope with the appropriate filters. Representative images were taken with a Nikon DS-Ri1 digital camera and edited in Adobe Photoshop.

Western blot analysis

Procedure was carried out as described previously [1]. Total protein extracts were obtained using a lysis buffer containing 30 mM Tris–HCl pH 7.5, 5 mM EDTA, 150 mM NaCl, 1% Triton X-100, 0.5% sodium deoxycolate, 0.1% SDS and 10% glycerol (1 hour at 4ºC; centrifugation at 13,000 rpm, 10 minutes, 4ºC). Protein concentration was measured with the BCA Protein Assay kit (Pierce). β-ACTIN is shown as a loading control. Antibodies were used at a 1:1000 dilution, except for β-ACTIN (1:3000). Images were processed with Adobe Photoshop CS5. A list of used primary and secondary antibodies in Supplementary table S6 online.

Flow cytometry analysis

Cells were trypsinized and resuspended in PBS. Cold methanol was added drop wise and incubated for 2 minutes at 4°C. After centrifugation (1,200 rpm, 5 minutes, 4ºC) pellet was resuspended in PBS and after another centrifugation in a blocking solution (PBS-BSA 1%, FBS 10%). After 30 minutes of incubation, samples were centrifuged and pellets were incubated in TGFβRI primary antibody (1:200 in a blocking solution, 20 minutes at 4°C). After primary antibody incubation, cells were washed with PBS and thereafter were incubated in 30 µL of secondary antibody (primary and secondary antibody source in Supplementary Table S6 online), 1:2000 for 30 minutes at 4ºC. After washing, cell fluorescence was analyzed in a Gallios flow cytometer (Kaluza version 1.1 software developed by Beckman Coulter).

Targeted know-down assays

SNU449 cell line was transfected with a combination of 4 shRNA plasmids for TGFβRI as well as a control unspecific shRNA. After 24 hours, media was changed to complete media, and selection of transfected cells was done with puromycin (for 50 days prior to experiments).

shRNA plasmids were selected from Mission SH (Sigma-Aldrich, St Louis, USA) with following sequences:

shTGFβRI:

#1 –CCGGCTCATGTTGATGGTCTATATCCTCGAGGATATAGACCATCAACATGAGTTTTTTG-

#2 –CCGGGAAGTTGCTGTTAAGATATTCCTCGAGGAATATCTTAACAGCAACTTCTTTTTTG-

#3 –CCGGGATCATGATTACTGTCGATAACTCGAGTTATCGACAGTAATCATGATCTTTTTTG-

#4 –CCGGGCTGGTCTTAACTTTAGGTAACTCGAGTTACCTAAAGTTAAGACCAGCTTTTTG-

Analysis of gene expression.

Real-time quantitative PCR reactions were performed using the following human specific primers designed by Integrated DNA Technologies (IDT):

L32	Forward: 5’-AACGTCAAGGAGCTGGAAG-3’
	Reverse: 5’-GGGTTGGTGACTCTGATGG-3’
TGFβ1	Forward: 5’-AAGTGGACATCAACGGGTTC-3’
	Reverse: 5’-GTCCTTGCGGAAGTCAATGT-3’

TGFβR1	Forward: 5’-ACATGATTCAGCCACAGATACC-3’
	Reverse: 5’-GCATAGATGTCAGCACGTTTG-3’
CDH1	Forward: 5’-CCCAATACATCTCCCTTCACAG-3’
	Reverse 5’-CCACCTCTAAGGCCATCTTTG-3’
CDH2	Forward: 5’-CCCAAGACAAAGAGACCCAG-3’
	Reverse: 5’-GCCACTGTGCTTACTGAATTG-3’
VIM	Forward: 5’- GGAAGCCTAACTACAGCGAG -3’
	Reverse: 5’- CAGAGTCCCAGATGAGCATTG -3’
SNAI1	Forward: 5′-GCTGCAGGACTCTAATCCAGAGTT-3′
	Reverse: 5’-GACAGAGTCCCAGATGAGCATTG-3’
TWIST1	Forward: 5′-CTCAGCTACGCCTTCTCG-3′
	Reverse: 5’-ACTGTCCATTTTCTCCTTCTCTG-3’
LDHA	Forward: 5’- GCAGCCTTTTCCTTAGAACAC -3’
	Reverse: 5’- AGATGTTCACGTTACGCTGG -3’
LDHB	Forward: 5’- CTTGCTCTTGTGGATGTTTTGG -3’
	Reverse: 5’- TCTTAGAATTGGCGGTCACAG -3’
PKM2	Forward: 5’- ATCGTCCTCACCAAGTCTGG -3’
	Reverse: 5’- GAAGATGCCACGGTACAGGT -3’
GLS1	Forward: 5’- GAAAGAGTACTGAGCCCTGAAG -3’
	Reverse: 5’- GGACAACTAAAAGAATGCCCC -3’
GLS2	Forward: 5’- TCCACAACTATGACAACCTGAG -3’
	Reverse: 5’- GCTGAGACATCGCCACTATAG -3’
SLC1A5	Forward: 5’- CCCTCATCTACTTCCTCTTCAC -3’
	Reverse: 5’- TTATTCTCCTCCACGCACTTC -3’
SLC7A5	Forward: 5’- TCTTCAACTGGCTCTGCG -3’
	Reverse: 5’- GAAGGAGACGGCGATCAG -3’
ACOX2	Forward: 5’- GAACATGCTGAGTCGCTTTG -3’
	Reverse: 5’- GGGAAGGTAGTTGCTCTGTG -3’

CPT2	Forward: 5'- TTGAGTGCTCCAAGTACCATG -3'
	Reverse: 5'- GCAAACAAGTGTCGGTCAAAG -3'
ACACA	Forward: 5'- CTGGAGGTGTATGTTCGAAGG -3'
	Reverse: 5'- TCTGTTTAGCGTAGGGATGTTC -3'
ACACB	Forward: 5'- CCCCAGACAAGTATCCCAAAG -3'
	Reverse: 5'- GGGTACTCCTGGGTCTTAAAC-3'
ACSL5	Forward: 5'- GTGCCTCGACTCCTTAACAG -3'
	Reverse: 5'- TGTCCCAGAAACTATCATGCC -3'
PPARγ	Forward: 5'- TTCTCCAGCATTTCTACTCCAC -3'
	Reverse: 5'- GCAGGCTCCACTTTGATTG -3'

Seahorse analysis

For the XF Cell Mito Stress analysis, ATP synthase inhibitor oligomycin (1μM) was injected to block proton movement and therefore oxidative phosphorylation. Consequently, carbonyl cyanide-*4*-(trifluoromethoxy) phenylhydrazone (FCCP, 1 μM) re-establishes proton movement by disrupting the mitochondrial membrane potential to stimulate maximal OCR and electron transport. At last, a combination of rotenone (1 μM) and antimycin (1 μM) was added to block complex I and III respectively and therefore completely block the mitochondrial respiration. The values obtained after injection of rotenone and antimycin were considered as non-mitochondrial respiration. The basal OCR was calculated as the last OCR measurement prior the addition of oligomycin minus the last OCR measurement after the addition of antimycin and rotenone. The ATP-linked OCR was calculated as the last OCR measurement prior oligomycin injection minus the last OCR measurement after the addition of oligomycin. The maximal OCR was calculated as the last OCR measurement after FCCP injection minus the last OCR measurement after the addition of antimycin and rotenone.

For the XF Glycolysis stress kit, glucose (10 mM) was injected, followed by oligomycin (1 μM), which as an ATP inhibitor permits the readout of the maximal ECAR. Finally, 2-DG (50 mM) was added to completely block glycolysis. The level of glycolysis is calculated as the last

ECAR measurement after glucose injection minus the last ECAR measurement after the addition of 2-DG. The glycolytic capacity is calculated as the last ECAR measurement after oligomycin injection minus the last ECAR measurement after 2-DG injection. The glycolytic reserve is calculated as the glycolytic capacity minus glycolysis.

Lactate production assay

The concentration of L-lactate was determined using an enzymatic reaction based on the oxidation of L-lactate to pyruvate by lactate dehydrogenase (5 mg of the enzyme (Roche, Basel, Switzerland)/ml, 550 U/mg) in the presence of NAD^+ (Sigma Aldrich) as previously described [2]. In this assay, the amount of NADH produced in the reaction is proportional to the amount of L-lactate in the samples. With this enzymatic system, D-lactate is not detected. All experiments were carried out at least 3 times in triplicates. The collected culture medium were diluted 1:20 with reaction mix [0.3 M hydrazine sulfate (Sigma Aldrich) and 0.87 M glycine (Sigma Aldrich)], pH 9.5; 2.5 M NAD^+ (Sigma Aldrich) and 0.19 M EDTA (Sigma Aldrich). Lactate dehydrogenase was added at a final concentration of 6.9 U/ml. The NADH concentration was determined as absorbance at 340 nm on a plate reader after 0 and 20 minutes after the start of the reaction. After the subtraction of absorbance at time 0, the absorbance at 20 minutes was normalization to L-lactate standards [(a serial dilution of sodium L-lactate (Sigma Aldrich)], obtained L-lactate concentrations were normalized to respective cell numbers.

Glucose consumption assay

The concentration of glucose was determined using a glucose oxidase and peroxidase method, PGO Enzymes (Sigma Aldrich) according to manufacturer's instructions. Briefly, to prepare the PGO enzyme reaction solution, 100 ml PGO Enzymes solution (1 PGO capsule in 100 ml of distilled water) was mixed with 1.6 ml of the o-Dianisidine solution [50 mg of o-Dianisidine hydrochloride (Sigma Aldrich) in 20 ml of distilled water]. Medium samples were diluted (1/20 in distilled water) and 15µl of sample was added to 200 µl of prepared reaction mix. After 30 minutes incubation at 37°C, absorbance was read on a plate reader at 450 nm. Different concentrations of glucose solution (Sigma Aldrich) served as the standards. The glucose consumption was calculated as the glucose concentration of medium control (no cells) minus

the glucose concentration of individual samples. All experiments were carried out at least 3 times in triplicates.

References

1 Bertran, E. *et al.* Overactivation of the TGF-β pathway confers a mesenchymal-like phenotype and CXCR4-dependent migratory properties to liver tumor cells. *Hepatology* **58(6)**, 2032-2044 (2013).

2 Álvarez, Z., Hyroššová, P., Perales, J. & Alcántara, S. Neuronal Progenitor Maintenance Requires Lactate Metabolism and PEPCK-M-Directed Cataplerosis. *Cereb Cortex* **26(3)**, 1046-1058 (2016).

Supplementary Fig. S1

a.

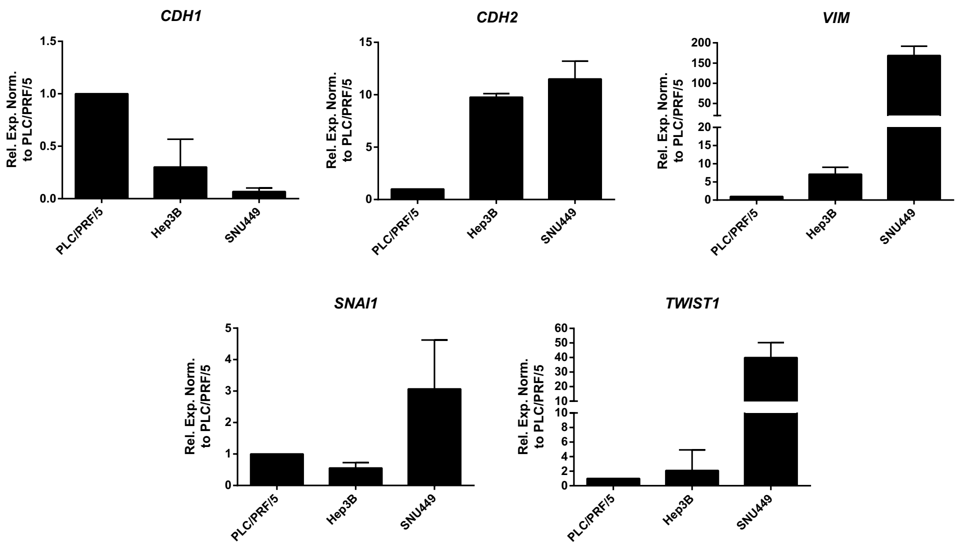

b.

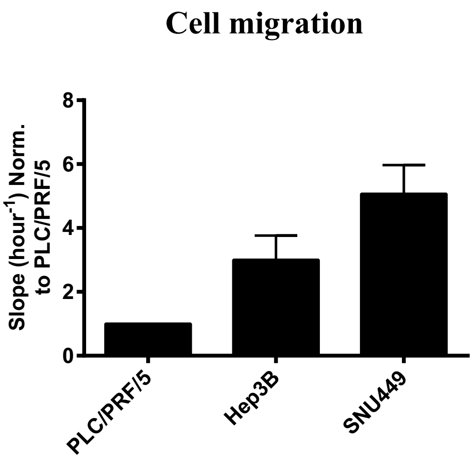

Characterization of HCC cell lines used in the study

(a) mRNA expression levels of EMT-related genes (*CDH1*, *CDH2* and *VIM*) and EMT-transcription factors (*SNAI1*, *TWIST1*) in three representative cell lines. qRT-PCR analysis normalized to PLC/PRF/5. Mean±SD (n=3). **(b)** Quantification as a slope (hour^{-1}) of the first 8 hours of cell migration in three representative cell lines normalised to PLC/PRF/5. Mean±SD (n=3).

Supplementary Fig. S2

a. **b.**

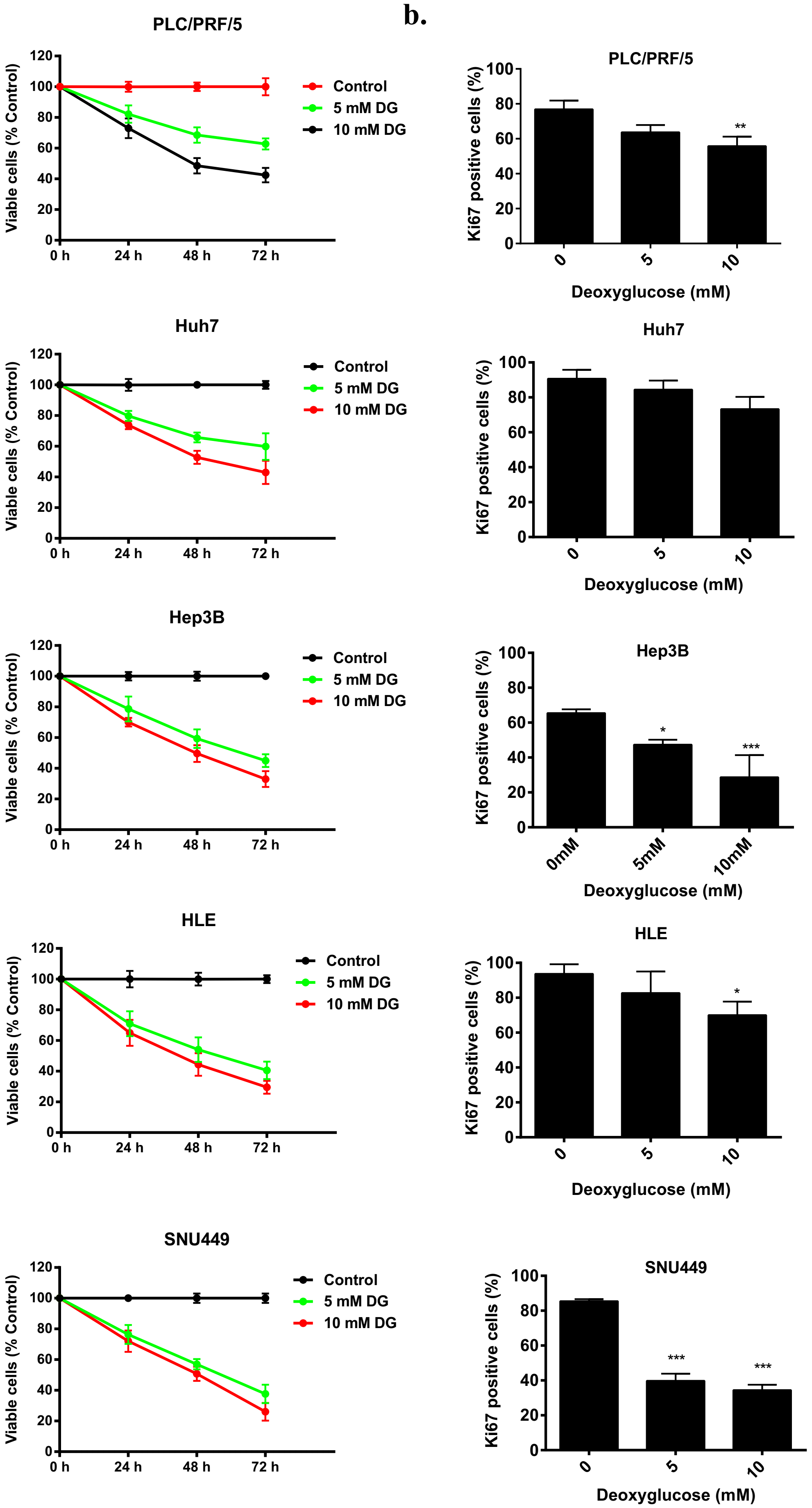

Response of HCC cell lines to deoxyglucose

PLC/PRF/5, Huh7, Hep3B, HLE and SNU449 cells were treated with deoxyglucose (0-10 mM).

(a) Cell viability was analyzed by crystal violet staining after 24, 48 and 72 hours of treatment and compared to un-treated control.

(b) Proliferation capacity was analyzed as the % of Ki67-positive cells after 72 hours of treatment.

Mean±SD (n=3). *$p< 0.05$, **$p<0.01$, ***$p<0.001$ as compared to un-treated control.

Supplementary Fig. S3

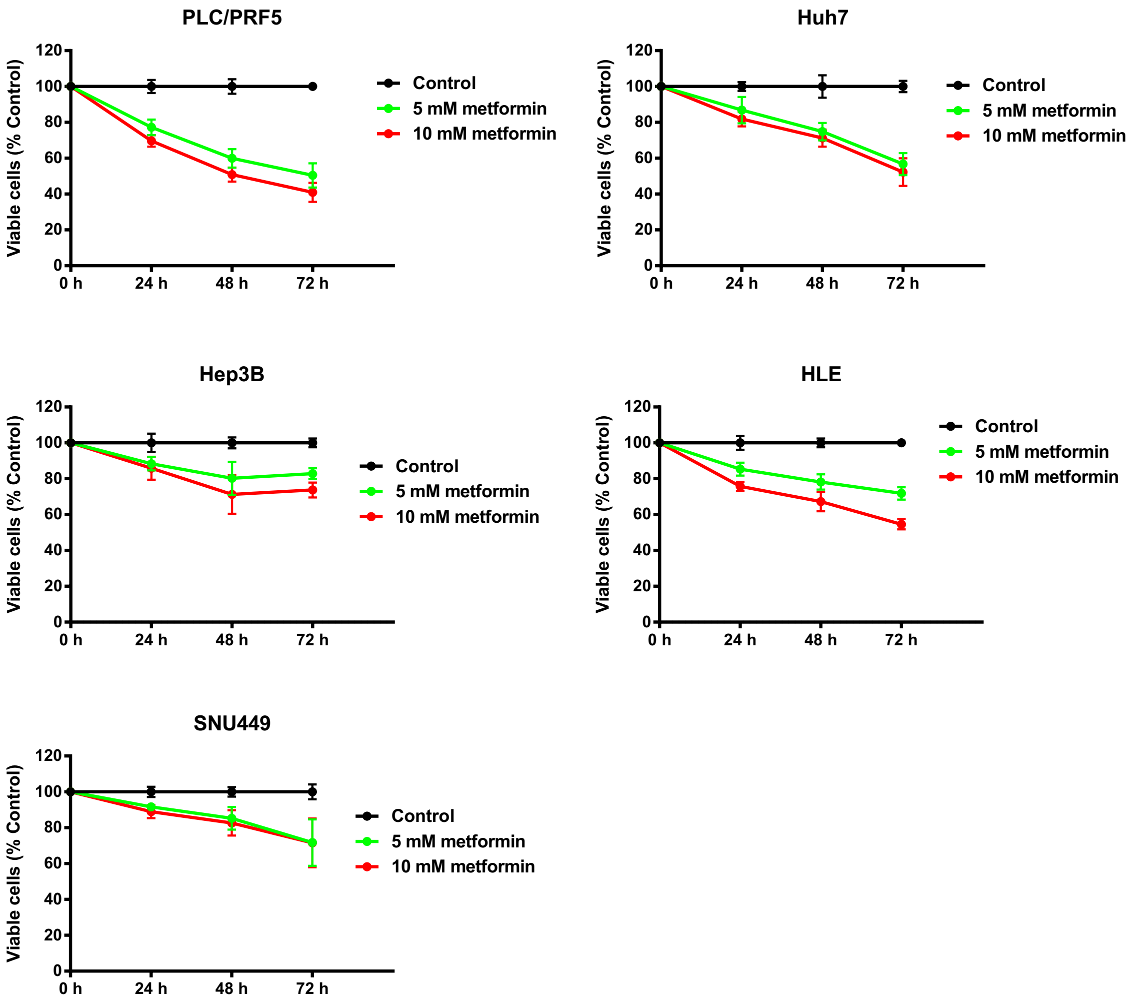

Response of HCC cell lines to metformin

PLC/PRF/5, Huh7, Hep3B, HLE and SNU449 cells were treated with metformin (0-10 mM). Cell viability was analyzed by crystal violet staining after 24, 48 and 72 hours of treatment and compared to un-treated control.

Supplementary Fig. S4

Sub Pathway	Biochemical Name	SNU449 / PLC/PRF/5	p value
Medium Chain Fatty Acid	laurate (12:0)	1.61	0.0008
Long Chain Fatty Acid	myristate (14:0)	2.04	0.0002
	myristoleate (14:1n5)	1.82	0.0070
	palmitoleate (16:1n7)	2.08	0.0132
	margarate (17:0)	1.54	0.0017
	10-heptadecenoate (17:1n7)	1.96	0.0009
	stearate (18:0)	1.30	0.0068
	nonadecanoate (19:0)	1.54	0.0069
	10-nonadecenoate (19:1n9)	4.17	0.0000
	arachidate (20:0)	2.08	0.0000
	eicosenoate (20:1)	3.85	0.0000
	tricosenoate (23:1)	2.13	0.0011
	erucate (22:1n9)	2.38	0.0003
	oleate/vaccenate (18:1)	2.33	0.0004
Polyunsaturated Fatty Acid (n3 and n6)	stearidonate (18:4n3)	2.13	0.0084
	docosapentaenoate (n3 DPA; 22:5n3)	2.44	0.0372
	docosatrienoate (22:3n3)	3.70	0.0048
	linoleate (18:2n6)	1.59	0.0376
	linolenate [alpha or gamma; (18:3n3 or 6)]	1.67	0.0444
	adrenate (22:4n6)	2.38	0.0312
	docosapentaenoate (n6 DPA; 22:5n6)	4.55	0.0113
	docosadienoate (22:2n6)	2.86	0.0000
	dihomo-linoleate (20:2n6)	5.26	0.0004
	mead acid (20:3n9)	5.56	0.0027
Fatty Acid, Branched	13-methylmyristate	1.92	0.0023
	15-methylpalmitate	1.85	0.0001
	17-methylstearate	1.82	0.0020

Sub Pathway	Biochemical Name	SNU449 / PLC/PRF/5	p value
Sphingolipid Metabolism	sphinganine	4.35	0.0003
	behenoyl sphingomyelin (d18:1/22:0)	2.04	0.0000
	sphingomyelin (d18:1/22:1, d18:2/22:0, d16:1/24:1)	1.69	0.0001
	sphingomyelin (d18:1/20:0, d16:1/22:0)	1.47	0.0016
	palmitoyl dihydrosphingomyelin (d18:0/16:0)	2.56	0.0004
	sphingomyelin (d18:1/24:1, d18:2/24:0)	1.79	0.0002
	sphingomyelin (d18:2/23:0, d18:1/23:1, d17:1/24:1)	1.89	0.0003
	sphingomyelin (d18:2/24:1, d18:1/24:2)	1.27	0.0016
	tricosanoyl sphingomyelin (d18:1/23:0)	1.64	0.0000
	palmitoyl sphingomyelin (d18:1/16:0)	1.41	0.0000
	phytosphingosine	6.25	0.0000

Sub Pathway	Biochemical Name	SNU449 / PLC/PRF/5	p value
Phospholipid Metabolism	1,2-dipalmitoyl-GPC (16:0/16:0)	1.18	0.0240
	1-palmitoyl-2-arachidonoyl-GPI (16:0/20:4)	1.37	0.0365
	1,2-dioleoyl-GPS (18:1/18:1)	2.56	0.0050
	1-palmitoyl-2-gamma-linolenoyl-GPC (16:0/18:3n6)	1.41	0.0156
	glycerophosphoethanolamine	7.14	0.0005
	glycerophosphoinositol	1.54	0.0017
	1-palmitoyl-2-oleoyl-GPC (16:0/18:1)	1.16	0.0008
	1-stearoyl-2-oleoyl-GPC (18:0/18:1)	1.37	0.0002
	1,2-dioleoyl-GPC (18:1/18:1)	1.41	0.0112

Differences in lipid metabolism between SNU449 and PLC/PRF/5 cells
List of detected metabolites. Statistical comparison of fold-changes between groups (SNU449/[PLC/PRF/5]) was performed by Welch's two-sample t-test. $p<0.05$ was considered significant. Values >1 indicate higher levels (red shading colour when significant) as compared to PLC/PRF/5. p value indicated in the right column.

Supplementary Fig. S5

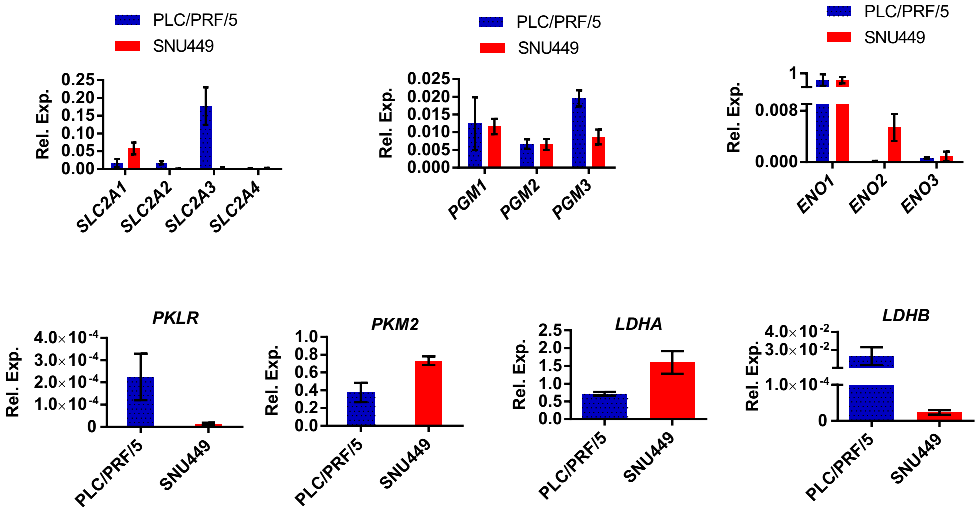

Relative expression of selected genes related to the glycolytic pathway in PLC/PRF/5 and SNU449 cells

mRNA expression levels were detected by qRT-PCR. Mean±SD (n=3).

Supplementary Fig. S6

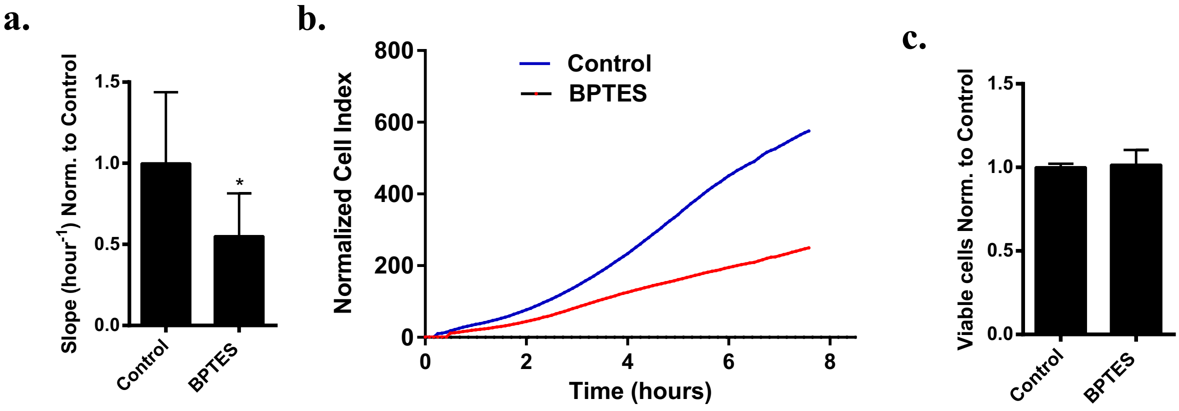

Analysis of cell migration in SNU449 cells after BPTES treatment

(a,b) SNU449 cells were treated with a GLS1 inhibitor BPTES (10 μM) for 12 hours. Cell migration was analyzed by real time xCELLigence system. **(a)** Quantification as a slope (hour^{-1}) of the first 8 hours of cell migration normalized to control condition. Mean±SD (n=8). *p<0.05. **(b)** A representative plot of cell migration over 8 hours. **(c)** Viable cells were analyzed by crystal violet staining after 24 hours of BPTES (10 μM) treatment and are normalized to control conditions.

Supplementary Fig. S7

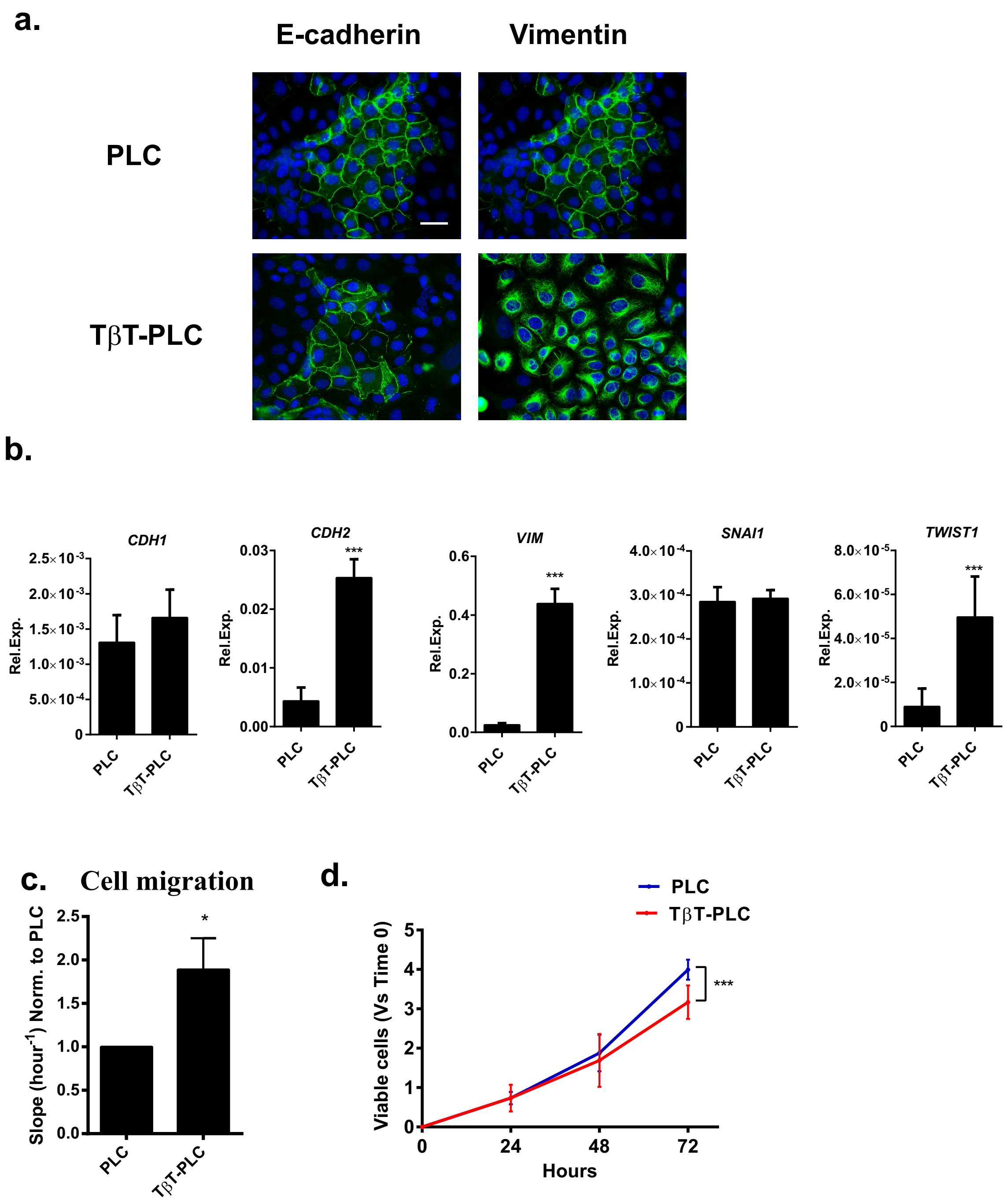

Generation of TGF-β treated PLC/PRF/5 cells (TβT-PLC)
PLC/PRF/5 cells were treated with TGF-β (2 ng/ml) for two weeks. Media was replaced every two days.
(a) Immunofluorescence analysis of E-cadherin (green) and vimentin (green). DAPI (blue). Scale bar represents 50 µm.
(b) mRNA expression levels of EMT-related genes (*CDH1, CDH2, VIM*) and EMT-inducing transcription factors (*SNAI1, TWIST1*) were detected by qRT-PCR and normalized to housekeeping gene *L32*. Mean±SD (n=3). *p<0.05, ***p<0.001 as compared to PLC.
(c) Quantification as a slope ($hour^{-1}$) of the first 8 hours of cell migration normalized to PLC. **(d)** Cell viability was analyzed by crystal violet staining after 24, 48 and 72 hours and normalized to time 0. Mean±SD (n=3). ***p<0.001 as compared to PLC.

Supplementary Fig. S8

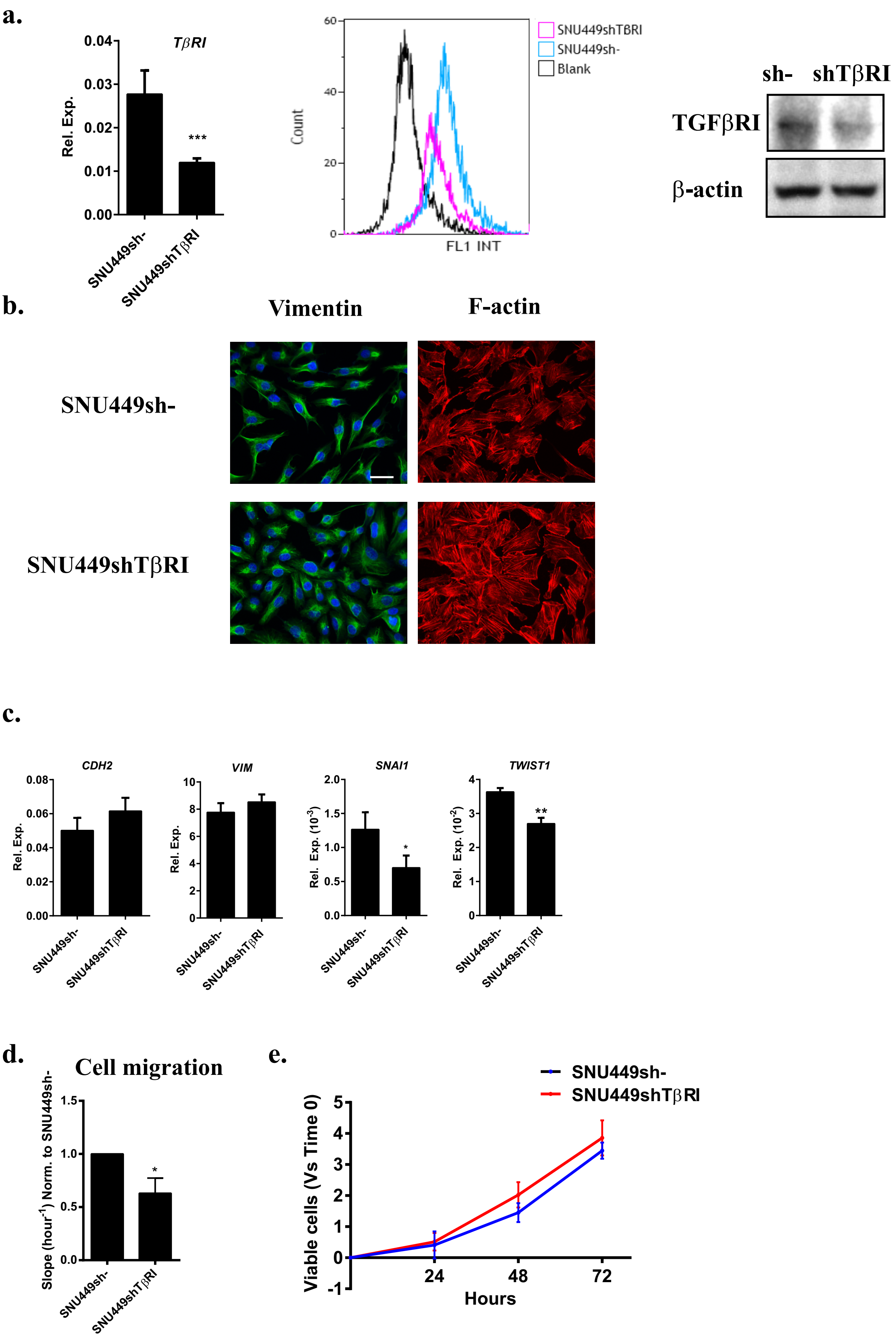

Stable knock down of TβRI in SNU449 cells

(a) mRNA expression levels of TβRI were detected by qRT-PCR and normalized to L32. Mean±SD (n=3). ***p<0.001 as compared to SNU449sh-. A representative FACS analysis of TGFβRI. Western blot analysis of TGFβRI. β-actin was used as a loading control. **(b)** Immunofluorescence analysis of Vimentin (green) and F-actin (red). DAPI (blue). Scale bar represents 25 µm. **(c)** mRNA expression levels of EMT-related genes (*CDH2, VIM*) and EMT-inducing transcription factors (*SNAI1, TWIST1*) were detected by qRT-PCR and normalized to housekeeping gene *L32*. Mean±SD (n=3).*p<0.05, **p<0.01 as compared to SNU449sh-. **(d)** Quantification as a slope (hour^{-1}) of the first 8 hours of cell migration normalized to SNU449sh-. Mean±SD (n=3). *p< 0.05 as compared to SNU449sh-. **(e)** Cell viability was analysed by crystal violet staining after 24, 48 and 72 hours and normalized to time 0. Mean±SD (n=3).

Supplementary Fig. S9

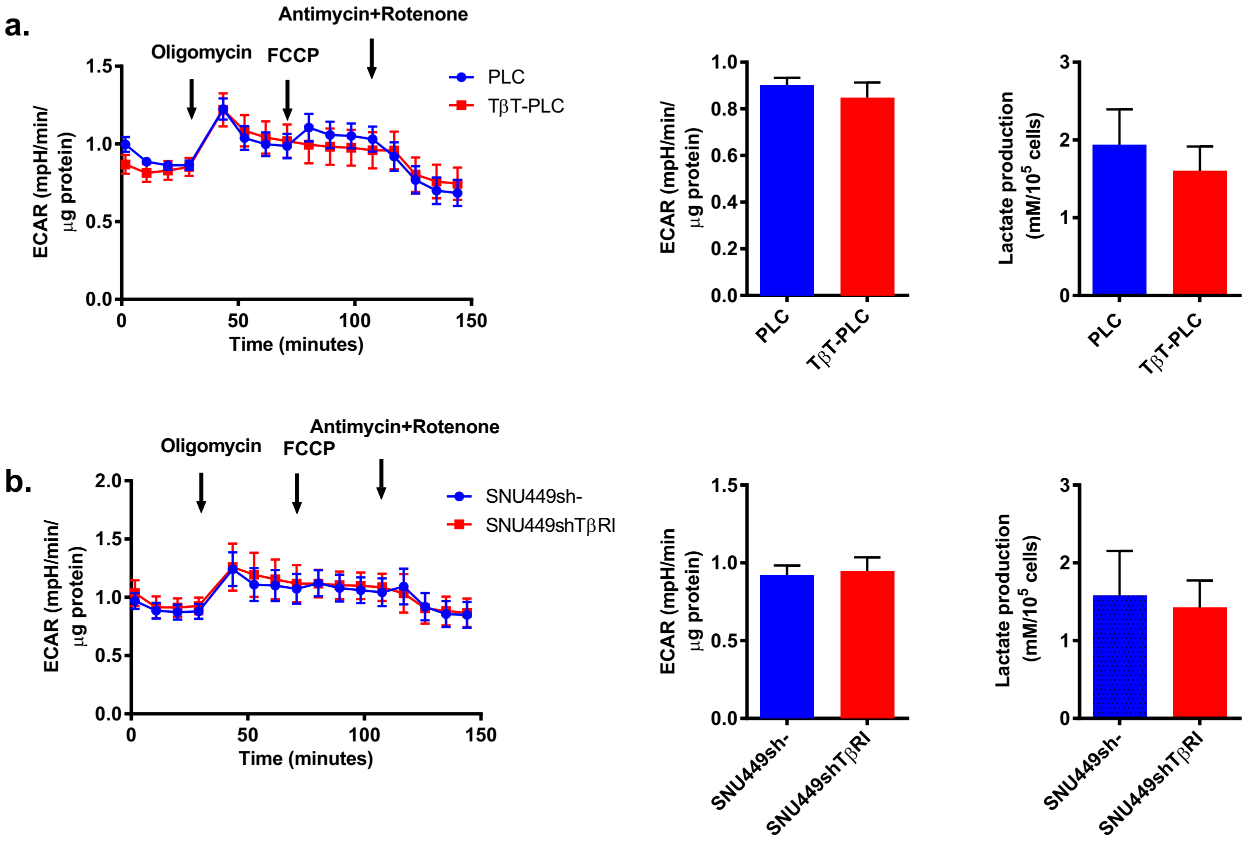

Analysis of the level of glycolysis in TβT-PLC/PLC and SNU449sh-/SNU449shTβRI cells

(a,b) ECAR normalized to protein content of PLC, TβT-PLC **(a)** and SNU449sh-, SNU449shTβRI cells **(b)** incubated 30 minutes prior experiment in XF assay medium supplemented with 5 mM glucose and 2 mM glutamine and consecutively injected with oligomycin (1 µM), FCCP (1.5 µM), antimycin (1µM) and rotenone (1 µM). Continuous ECAR values (pmoles/min/µg protein) are shown. Basal ECAR was measured before the injection of mitochondrial stress inhibitors. Mean±SEM (n at least 6 from three independent experiments). Lactate production (mM/10^5 cells) was measured after 48 hours of cultivation in DMEM supplemented with 10% FBS and normalized to cell number. Mean±SD (n=3).

Supplementary Fig. S10

a.

Statistical Comparisons
Welch's Two-Sample *t*-Test

Significantly Altered Biochemicals	TβT-PLC PLC	SNU449sh- SNU449shTβRI
Total biochemicals $p \leq 0.05$	107	147
Biochemicals (↑↓)	55|52	49|98
Total biochemicals $0.05 < p < 0.10$	41	46
Biochemicals (↑↓)	21|20	15|31

b.

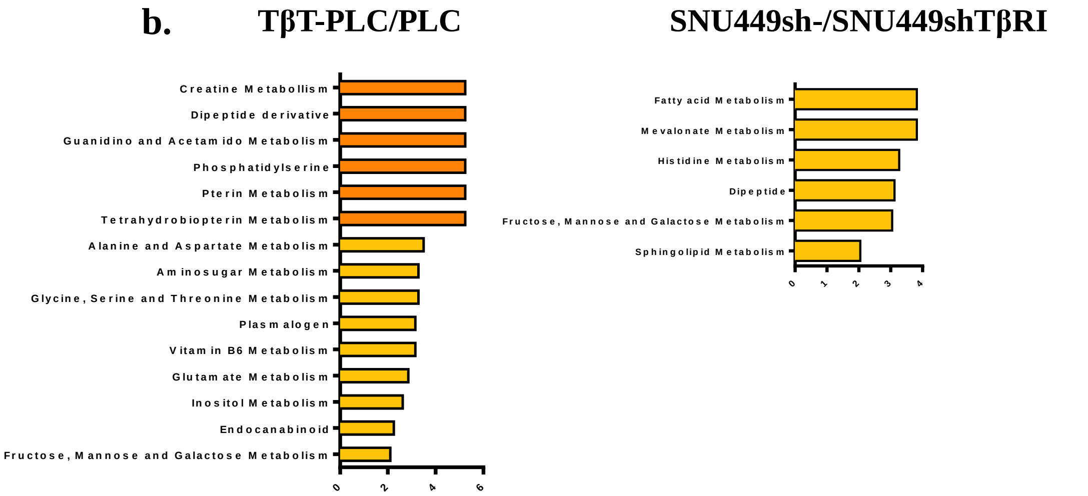

c.

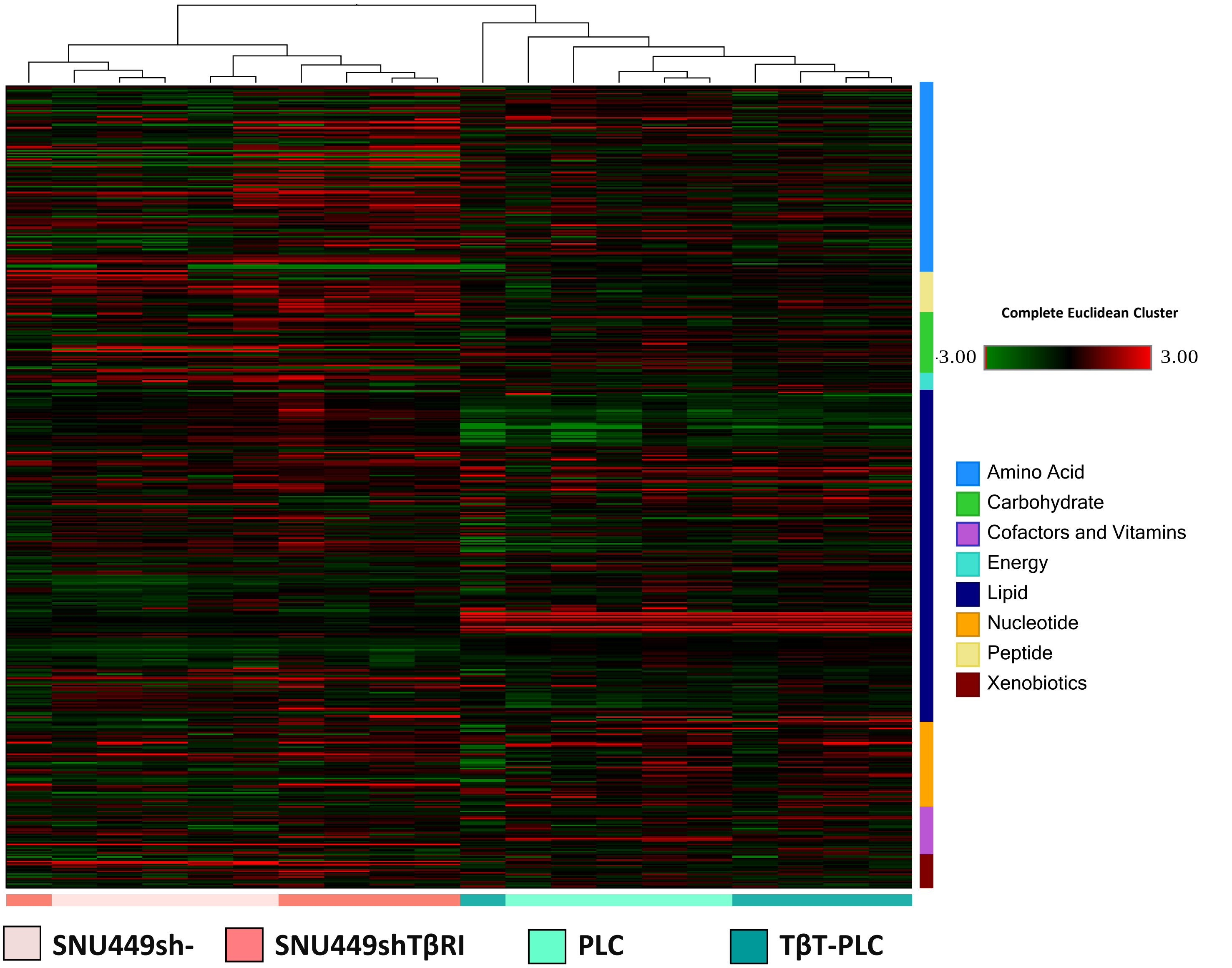

Metabolomic analysis of TβT-PLC/PLC and SNU449sh-/SNU449shTβRI cells
(a) A total of 561 metabolites were detected. Welch's two-sample *t*-test was used to identify biochemicals that differed significantly ($p<0.05$) and with approaching significance ($0.05<p>0.10$) between experimental groups (n=5 for each group). **(b)** Pathway set enrichment analysis was performed using MetaboLync[TM] platform and followed formula: # of significant metabolites in pathway (k) / total # of detected metabolites in pathway (m)) / [total # of significant metabolites (n) / total # of detected metabolites(N)] (k/m)/(n/N). **(c)** Hierarchical clustering was performed using Euclidean distance method by Metabolon Inc.

Supplementary Fig. S11

a.

Sub Pathway	Biochemical Name	SNU449sh-/SNU449shTβRI	p value
Sphingolipid Metabolism	N-palmitoyl-sphinganine (d18:0/16:0)	1.72	0.0266
	sphinganine	1.52	0.0056
	sphingomyelin (d18:1/24:1, d18:2/24:0)	1.28	0.0288
	behenoyl sphingomyelin (d18:1/22:0)	1.56	0.0003
	sphingomyelin (d18:1/22:1, d18:2/22:0, d16:1/24:1)	1.64	0.0002
	sphingomyelin (d18:1/20:0, d16:1/22:0)	1.61	0.0001
	palmitoyl dihydrosphingomyelin (d18:0/16:0)	1.49	0.0324
	sphingomyelin (d18:1/21:0, d17:1/22:0, d16:1/23:0)	1.72	0.0001
	sphingomyelin (d18:2/23:0, d18:1/23:1, d17:1/24:1)	1.35	0.0007
	sphingomyelin (d18:2/24:1, d18:1/24:2)	1.27	0.0106
	tricosanoyl sphingomyelin (d18:1/23:0)	1.18	0.0340

b.

Sub Pathway	Biochemical Name	SNU449sh-/SNU449shTβRI	p value
Phospholipid Metabolism	choline phosphate	1.35	0.0266
	1,2-dipalmitoyl-GPC (16:0/16:0)	1.32	0.0006
	1-stearoyl-2-arachidonoyl-GPC (18:0/20:4)	1.41	0.0114
	1-palmitoyl-2-arachidonoyl-GPC (16:0/20:4)	1.28	0.0115
	1-palmitoyl-2-arachidonoyl-GPI (16:0/20:4)	1.54	0.0002
	1,2-dioleoyl-GPS (18:1/18:1)	1.39	0.0353
	1-stearoyl-2-linoleoyl-GPS (18:0/18:2)	1.25	0.0128
	1-palmitoyl-2-gamma-linolenoyl-GPC (16:0/18:3n6)	1.41	0.0411

c.

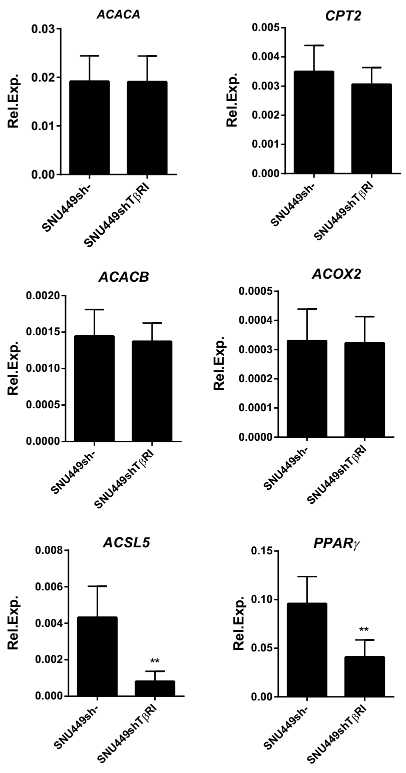

Analysis of lipid content and expression of genes related to fatty acid synthesis in SNU449sh-/SNU449shTβRI cells
(a,b) Metabolites from the sphingolipid **(a)** and phospholipid **(b)** metabolism pathway presented in fold. Welch's two-sample t-test was used to identify metabolites that differed significantly between experimental groups (n=5 for each group, p value indicated in the right column). **(c)** Relative expression of selected genes related to fatty acid β-oxidation and fatty acid synthesis detected by qRT-PCR. Mean±SD. (n=3). **p<0.01.

Supplementary Table S1

List of detected metabolites in PLC/PRF/5 compared to SNU449 cells

Sub Pathway	Biochemical Name	SNU449 / PLC/PRF/5	p value
Glycolysis, Gluconeogenesis, and Pyruvate Metabolism	glucose	0.57	0.2056
	glucose 6-phosphate	**0.20**	0.0011
	fructose-6-phosphate	1.32	0.3476
	Isobar: fructose 1,6-diphosphate, glucose 1,6-diphosphate, myo-inositol 1,4 or 1,3-diphosphate	**25.00**	0.0005
	dihydroxyacetone phosphate (DHAP)	**5.26**	0.0054
	3-phosphoglycerate	2.50	0.1829
	phosphoenolpyruvate (PEP)	1.89	0.1953
	pyruvate	**1.89**	0.0000
	lactate	1.06	0.5223
	glycerate	3.70	0.1742
Pentose Phosphate Pathway	6-phosphogluconate	0.95	0.5611
	ribose 1-phosphate	0.62	0.0921
	sedoheptulose-7-phosphate	**0.42**	0.0020
Pentose Metabolism	ribose	0.35	0.0610
	ribitol	**4.76**	0.0000
	ribonate	**0.10**	0.0000
	arabitol/xylitol	0.73	0.2858
	ribulose/xylulose	**0.15**	0.0023
	arabonate/xylonate	**0.23**	0.0002
Glycogen Metabolism	maltotriose	0.63	0.3739
	maltose	0.75	0.2605
Disaccharides and Oligosaccharides	sucrose	1.16	0.1044
Fructose, Mannose and Galactose Metabolism	fructose	**5.56**	0.0037
	mannitol/sorbitol	**7.69**	0.0000
	mannose	**0.20**	0.0007
	galactitol (dulcitol)	**9.09**	0.0000
	galactonate	**2.38**	0.0316
Nucleotide Sugar	UDP-glucose	**0.44**	0.0003
	UDP-galactose	**0.49**	0.0126
	UDP-glucuronate	**6.67**	0.0000
	guanosine 5'-diphospho-fucose	**0.72**	0.0003
	UDP-N-acetylglucosamine	1.25	0.2540
	cytidine 5'-monophospho-N-acetylneuraminic acid	0.85	0.0820
	glucuronate 1-phosphate*	**8.33**	0.0003
Aminosugar Metabolism	glucosamine-6-phosphate	**0.30**	0.0318
	glucuronate	**1.69**	0.0237
	N-acetylglucosamine 6-phosphate	**0.13**	0.0000
	N-acetyl-glucosamine 1-phosphate	**0.35**	0.0016
	N-acetylneuraminate	**3.57**	0.0000
	N-acetylglucosaminylasparagine	**1.64**	0.0061
	erythronate	**0.22**	0.0000
	N-acetylglucosamine/N-acetylgalactosamine	0.60	0.0835
	acetyl CoA	1.28	0.3259

TCA Cycle	citrate	6.67	0.0002
	aconitate [cis or trans]	4.17	0.0001
	isocitrate	5.00	0.0024
	alpha-ketoglutarate	1.82	0.0026
	succinylcarnitine	1.79	0.0880
	succinate	3.45	0.0224
	fumarate	0.90	0.3819
	malate	0.97	0.7900
	itaconate	1.20	0.0021
	2-methylcitrate/homocitrate	0.69	0.0001
Oxidative Phosphorylation	acetylphosphate	1.35	0.4616
	phosphate	0.37	0.0052
Glycine, Serine and Threonine Metabolism	glycine	1.59	0.0020
	N-acetylglycine	0.88	0.1453
	betaine	0.43	0.0007
	betaine aldehyde	0.07	0.0008
	serine	1.20	0.0490
	N-acetylserine	0.61	0.0006
	threonine	0.99	0.8056
	N-acetylthreonine	0.42	0.0000
Alanine and Aspartate Metabolism	alanine	1.15	0.1596
	N-acetylalanine	0.72	0.0032
	aspartate	0.62	0.0004
	asparagine	0.46	0.0001
	N-acetylasparagine	0.05	0.0000
	N-acetylaspartate (NAA)	1.20	0.1266
Glutamate Metabolism	glutamate	0.93	0.1438
	glutamine	0.91	0.2565
	N-acetylglutamate	0.99	0.8211
	N-acetylglutamine	0.08	0.0000
	N-acetyl-aspartyl-glutamate (NAAG)	1.23	0.1027
	gamma-aminobutyrate (GABA)	0.63	0.0534
	4-hydroxyglutamate	0.05	0.0000
	glutamate, gamma-methyl ester	0.78	0.2574
	pyroglutamine	0.44	0.0009
	gamma-carboxyglutamate	0.83	0.4345
	S-1-pyrroline-5-carboxylate	2.44	0.0013
Histidine Metabolism	histidine	1.19	0.0487
	N-acetylhistidine	0.30	0.0000
	1-methylhistidine	0.23	0.0026
	imidazole propionate	0.71	0.1290
	imidazole lactate	0.98	0.7411
	4-imidazoleacetate	1.61	0.0144
	histidine methyl ester	0.49	0.0003
Lysine Metabolism	lysine	1.28	0.0358
	N2-acetyllysine	0.88	0.3441
	N6-acetyllysine	0.69	0.2078
	N6,N6,N6-trimethyllysine	1.05	0.4550
	5-hydroxylysine	0.97	0.9090
	saccharopine	0.16	0.0000

Lysine Metabolism	2-aminoadipate	0.49	0.0007
	glutarate (pentanedioate)	0.63	0.0048
	3-hydroxyglutarate	0.52	0.0019
	pipecolate	1.43	0.0945
	cadaverine	1.22	0.0490
	N-acetyl-cadaverine	10.00	0.0002
Phenylalanine and Tyrosine Metabolism	phenylalanine	1.52	0.0007
	N-acetylphenylalanine	0.02	0.0000
	phenyllactate (PLA)	5.26	0.0000
	tyrosine	1.08	0.3310
	N-acetyltyrosine	0.05	0.0000
	4-hydroxyphenylpyruvate	0.80	0.3418
	3-(4-hydroxyphenyl)lactate	0.93	0.4090
	phenol sulfate	1.61	0.2860
	p-cresol sulfate	0.11	0.0000
Tryptophan Metabolism	tryptophan	1.35	0.0114
	N-acetyltryptophan	0.02	0.0000
	indolelactate	2.44	0.0187
	kynurenine	2.50	0.0020
	kynurenate	0.23	0.0043
	C-glycosyltryptophan	1.85	0.0059
	N-acetylkynurenine (2)	0.65	0.0534
	thioproline	1.59	0.0003
Leucine, Isoleucine and Valine Metabolism	leucine	1.22	0.0183
	N-acetylleucine	0.09	0.0000
	4-methyl-2-oxopentanoate	1.56	0.0161
	isovalerylcarnitine	0.35	0.0392
	beta-hydroxyisovalerate	1.06	0.7861
	alpha-hydroxyisovalerate	2.86	0.0002
	methylsuccinate	0.91	0.4656
	isoleucine	1.25	0.0105
	N-acetylisoleucine	0.19	0.0001
	3-methyl-2-oxobutyrate	4.76	0.0022
	3-methyl-2-oxovalerate	1.49	0.3739
	2-methylbutyrylcarnitine (C5)	1.08	0.8205
	tiglylcarnitine	2.08	0.1447
	2-hydroxy-3-methylvalerate	3.45	0.0001
	ethylmalonate	2.70	0.0000
	valine	1.30	0.0126
	N-acetylvaline	0.42	0.0000
	isobutyrylcarnitine	0.96	0.7039
	isobutyrylglycine	1.09	0.5439
	3-hydroxyisobutyrate	3.45	0.0014
	alpha-hydroxyisocaproate	10.00	0.0001
	methionine	1.11	0.2978
	N-acetylmethionine	0.76	0.0047
	N-formylmethionine	1.56	0.0285
	methionine sulfone	0.36	0.0083
	methionine sulfoxide	1.18	0.2208
	N-acetylmethionine sulfoxide	0.57	0.0151

Methionine, Cysteine, SAM and Taurine Metabolism	S-adenosylmethionine (SAM)	1.85	0.0010
	S-adenosylhomocysteine (SAH)	1.14	0.3899
	cystathionine	0.38	0.0000
	cysteine	2.94	0.0006
	N-acetylcysteine	1.10	0.4221
	cystine	9.09	0.0005
	S-methylcysteine	2.78	0.0060
	cysteine s-sulfate	7.14	0.0002
	cysteine sulfinic acid	0.38	0.0094
	hypotaurine	1.45	0.0311
	taurine	1.61	0.0454
	N-acetyltaurine	0.51	0.0164
	2-hydroxybutyrate/2-hydroxyisobutyrate	0.51	0.0015
Urea cycle; Arginine and Proline Metabolism	arginine	6.25	0.0062
	ornithine	0.31	0.0002
	proline	0.65	0.0004
	citrulline	0.04	0.0053
	argininosuccinate	0.27	0.0784
	homoarginine	0.67	0.0971
	homocitrulline	0.79	0.5428
	dimethylarginine (SDMA + ADMA)	0.75	0.0948
	N-acetylarginine	0.72	0.2036
	N-delta-acetylornithine	0.90	0.3829
	trans-4-hydroxyproline	1.15	0.1953
	pro-hydroxy-pro	0.41	0.0008
	N-monomethylarginine	0.67	0.1656
	N-acetylcitrulline	1.00	
Creatine Metabolism	creatine	0.36	0.0002
	creatinine	0.55	0.0003
	creatine phosphate	4.55	0.0035
	guanidinoacetate	5.26	0.0001
Polyamine Metabolism	putrescine	16.67	0.0001
	spermidine	3.57	0.0024
	5-methylthioadenosine (MTA)	4.17	0.0000
	N-acetylputrescine	6.67	0.0001
Guanidino and Acetamido Metabolism	4-guanidinobutanoate	1.12	0.3844
Glutathione Metabolism	glutathione, reduced (GSH)	0.26	0.0564
	glutathione, oxidized (GSSG)	0.27	0.0410
	cysteine-glutathione disulfide	0.85	0.2549
	S-methylglutathione	0.64	0.1014
	cysteinylglycine	4.55	0.3069
	5-oxoproline	1.54	0.0405
	ophthalmate	0.22	0.0180
Glycerolipid Metabolism	glycerol	9.09	0.0001
	glycerol 3-phosphate	7.69	0.0001
	glycerophosphoglycerol	1.14	0.2913

List of detected metabolites. Statistical comparison of fold-changes between groups (SNU449/[PLC/PRF/5]) was performed by Welch's two-sample t-test. $p<0.05$ was considered significant. Values <1 indicate lower levels (green shading colour when significant) and values >1 indicate higher levels (red shading colour when significant) as compared to PLC/PRF/5.

Supplementary Table S2

List of analysed genes in PLC/PRF/5 cells and compared to SNU449

Sub Pathway	Symbol	GenBank	Description	SNU449 / PLC/PRF/5	p-value
Glycolysis	***ALDOA***	NM_000034	Aldolase A, fructose-bisphosphate	0.63	0.5443
	ALDOB	NM_000035	Aldolase B, fructose-bisphosphate	N/A	N/A
	ALDOC	NM_005165	Aldolase C, fructose-bisphosphate	N/A	N/A
	BPGM	NM_001724	2,3-bisphosphoglycerate mutase	1.54	0.1976
	ENO1	NM_001428	Enolase 1, (alpha)	0.92	0.8920
	ENO2	NM_001975	Enolase 2 (gamma, neuronal)	**40.98**	0.0122
	ENO3	NM_001976	Enolase 3 (beta, muscle)	1.35	0.6261
	GALM	NM_138801	Galactose mutarotase (aldose 1-epimerase)	0.99	0.9795
	GCK	NM_000162	Glucokinase (hexokinase 4)	N/A	N/A
	GPI	NM_000175	Glucose-6-phosphate isomerase	1.10	0.8321
	HK2	NM_000189	Hexokinase 2	**0.05**	0.0154
	HK3	NM_002115	Hexokinase 3 (white cell)	N/A	N/A
	LDHA	NM_005566	Lactate dehydrogenase A	**2.22**	0.0090
	LDHB	NM_002300	Lactate dehydrogenase B	**0.00**	0.0007
	PFKL	NM_002626	Phosphofructokinase, liver	0.63	0.4835
	PGAM2	NM_000290	Phosphoglycerate mutase 2 (muscle)	0.76	0.6248
	PGK1	NM_000291	Phosphoglycerate kinase 1	0.86	0.6257
	PGK2	NM_138733	Phosphoglycerate kinase 2	0.62	0.4643
	PGM1	NM_002633	Phosphoglucomutase 1	0.94	0.8710
	PGM2	NM_018290	Phosphoglucomutase 2	0.98	0.9260
	PGM3	NM_015599	Phosphoglucomutase 3	**0.44**	0.0035
	PKLR	NM_000298	Pyruvate kinase, liver and RBC	**0.06**	0.0247
	PKM2	NM_002654	Pyruvate kinase, muscle 2	**1.96**	0.0095
	TPI1	NM_000365	Triosephosphate isomerase 1	1.06	0.8372
Pyruvate dehydrogenase regulation	***PDK1***	NM_002610	Pyruvate dehydrogenase kinase, isozyme 1	1.49	0.3387
	PDK2	NM_002611	Pyruvate dehydrogenase kinase, isozyme 2	0.61	0.3902
	PDK3	NM_005391	Pyruvate dehydrogenase kinase, isozyme 3	0.44	0.0980
	PDK4	NM_002612	Pyruvate dehydrogenase kinase, isozyme 4	0.61	0.3342
	PDP2	NM_020786	Pyruvate dehyrogenase phosphatase catalytic subunit 2	1.10	0.7999
	PDPR	NM_017990	Pyruvate dehydrogenase phosphatase regulatory subunit	1.02	0.9708
Pyruvate dehydrogenase complex	***DLAT***	NM_001931	Dihydrolipoamide S-acetyltransferase	**0.20**	0.0418
	DLD	NM_000108	Dihydrolipoamide dehydrogenase	1.50	0.1132
	DLST	NM_001933	Dihydrolipoamide S-succinyltransferase	0.91	0.8602
	PDHA1	NM_000284	Pyruvate dehydrogenase (lipoamide) alpha 1	**2.01**	0.0004
	PDHB	NM_000925	Pyruvate dehydrogenase (lipoamide) beta	0.65	0.1146
Gluconeogenesis	***FBP1***	NM_000507	Fructose-1,6-bisphosphatase 1	N/A	N/A
	FBP2	NM_003837	Fructose-1,6-bisphosphatase 2	0.71	0.1834
	G6PC	NM_000151	Glucose-6-phosphatase, catalytic subunit	0.25	0.2564
	G6PC3	NM_138387	Glucose 6 phosphatase, catalytic, 3	1.07	0.8950
	PC	NM_000920	Pyruvate carboxylase	0.32	0.1860
	PCK1	NM_002591	Phosphoenolpyruvate carboxykinase 1 (soluble)	N/A	N/A
	PCK2	NM_004563	Phosphoenolpyruvate carboxykinase 2	0.25	0.1085
	G6PD	NM_000402	Glucose-6-phosphate dehydrogenase	**6.20**	0.0006
	H6PD	NM_004285	Hexose-6-phosphate dehydrogenase (glucose 1-dehydrogenase)	0.44	0.2948
	PGLS	NM_012088	6-phosphogluconolactonase	0.57	0.2679

**Pentose Phosphate Pathway**	***PRPS1***	NM_002764	Phosphoribosyl pyrophosphate synthetase 1	0.58	0.2702
	PRPS1L1	NM_175886	Phosphoribosyl pyrophosphate synthetase 1-like 1	N/A	N/A
	PRPS2	NM_002765	Phosphoribosyl pyrophosphate synthetase 2	1.77	0.0682
	RBKS	NM_022128	Ribokinase	1.32	0.2307
	RPE	NM_199229	Ribulose-5-phosphate-3-epimerase	1.01	0.9764
	RPIA	NM_144563	Ribose 5-phosphate isomerase A	1.07	0.7885
	TALDO1	NM_006755	Transaldolase 1	1.21	0.5520
	TKT	NM_001064	Transketolase	**3.63**	0.0109
**Metabolism of glycogen**	***AGL***	NM_000028	Amylo-alpha-1, 6-glucosidase, 4-alpha-glucanotransferase	**0.15**	0.0187
	GBE1	NM_000158	Glucan (1,4-alpha-), branching enzyme 1	0.96	0.8878
	GSK3A	NM_019884	Glycogen synthase kinase 3 alpha	0.79	0.6949
	GSK3B	NM_002093	Glycogen synthase kinase 3 beta	1.57	0.1640
	GYS1	NM_002103	Glycogen synthase 1 (muscle)	1.22	0.7296
	GYS2	NM_021957	Glycogen synthase 2 (liver)	N/A	N/A
	PGM1	NM_002633	Phosphoglucomutase 1	0.94	0.8710
	PGM2	NM_018290	Phosphoglucomutase 2	0.98	0.9260
	PGM3	NM_015599	Phosphoglucomutase 3	**0.44**	0.0035
	PHKA1	NM_002637	Phosphorylase kinase, alpha 1 (muscle)	0.73	0.4795
	PHKB	NM_000293	Phosphorylase kinase, beta	1.54	0.0946
	PHKG1	NM_006213	Phosphorylase kinase, gamma 1 (muscle)	N/A	N/A
	PHKG2	NM_000294	Phosphorylase kinase, gamma 2 (testis)	1.28	0.4469
	PYGL	NM_002863	Phosphorylase, glycogen, liver	**0.54**	0.0271
	PYGM	NM_005609	Phosphorylase, glycogen, liver	N/A	N/A
	UGP2	NM_006759	UDP-glucose pyrophosphorylase 2	1.15	0.3779
**TCA cycle**	***ACLY***	NM_001096	ATP citrate lyase	1.39	0.4303
	ACO1	NM_002197	Aconitase 1, soluble	1.14	0.6138
	ACO2	NM_001098	Aconitase 2, mitochondrial	0.73	0.5335
	CS	NM_004077	Citrate synthase	0.98	0.9663
	FH	NM_000143	Fumarate hydratase	**2.18**	0.0101
	IDH1	NM_005896	Isocitrate dehydrogenase 1 (NADP+), soluble	0.79	0.3012
	IDH2	NM_002168	Isocitrate dehydrogenase 2 (NADP+), mitochondrial	0.34	0.2045
	IDH3A	NM_005530	Isocitrate dehydrogenase 3 (NAD+) alpha	0.81	0.4194
	IDH3B	NM_174856	Isocitrate dehydrogenase 3 (NAD+) beta	0.85	0.7443
	IDH3G	NM_174869	Isocitrate dehydrogenase 3 (NAD+) gamma	2.03	0.1372
	MDH1	NM_005917	Malate dehydrogenase 1, NAD (soluble)	0.96	0.7999
	MDH1B	NM_00103984	Malate dehydrogenase 1B, NAD (soluble)	N/A	N/A
	MDH2	NM_005918	Malate dehydrogenase 2, NAD (mitochondrial)	0.72	0.4761
	OGDH	NM_002541	Oxoglutarate dehydrogenase	1.33	0.4895
	PC	NM_000920	Pyruvate carboxylase	0.32	0.1860
	PCK1	NM_002591	Phosphoenolpyruvate carboxykinase 1 (soluble)	N/A	N/A
	PCK2	NM_004563	Phosphoenolpyruvate carboxykinase 2	0.25	0.1085
	SDHA	NM_004168	Succinate dehydrogenase complex, subunit A, flavoprotein (Fp)	0.59	0.3254
	SDHB	NM_003000	Succinate dehydrogenase complex, subunit B, iron sulfur (Ip)	1.06	0.7323
	SDHC	NM_003001	Succinate dehydrogenase complex, subunit C, integral membrane protein	0.60	0.1640
	SDHD	NM_003001	Succinate dehydrogenase complex, subunit D, integral membrane protein	1.00	0.9888
	SUCLA2	NM_003850	Succinate-CoA ligase, ADP-forming, beta subunit	1.09	0.7805
	SUCLG1	NM_003849	Succinate-CoA ligase, alpha subunit	1.15	0.2048

	SUCLG2	NM_003848	Succinate-CoA ligase, GDP-forming, beta subunit	0.64	0.3147
**Glutamine Metabolism**	***GLS1***	NM_014905	Glutaminase 1	**2.33**	0.0025
	GLS2	NM_013267	Glutaminase 2	N/A	N/A
	SLC1A5	NM_005628	Solute carrier family 1 (neutral amino acid transporter), member 5	1.70	0.1222
	SLC7A5	NM_003486	Solute carrier family 7 (amino acid transporter light chain, L system), member 5	**5.32**	0.0001

List of analysed genes. Expression in fold comparing PLC/PRF/5 to SNU449. $p<0.05$ was considered significant. Values <1 indicate lower expression (green shading colour when significant) and values >1 indicate higher expression (red shading colour when significant) as compared to PLC/PRF/5. N/A indicates Ct values >35 and considered as non-detectable gene expression.

Supplementary Table S3

List of detected metabolites in PLC vs TβT-PLC and in SNU449sh- vs SNU449shTβRI cells

Sub Pathway	Biochemical Name	TβT-PLC/ PLC	p value	SNU449sh-/ SNU449shTβRI	p value
Glycolysis, Gluconeogenesis, and Pyruvate Metabolism	glucose	0.62	0.2924	1.11	0.6975
	glucose 6-phosphate	0.41	0.0135	1.02	0.8179
	fructose-6-phosphate	0.73	0.7185	0.79	0.1957
	Isobar: fructose 1,6-diphosphate, glucose 1,6-diphosphate, myo-inositol 1,4 or 1,3-diphosphate	1.46	0.4133	0.64	0.1979
	dihydroxyacetone phosphate (DHAP)	0.93	0.5186	0.80	0.3273
	3-phosphoglycerate	1.71	0.0727	1.64	0.5601
	phosphoenolpyruvate (PEP)	1.32	0.2740	1.37	0.6112
	pyruvate	1.09	0.0479	1.16	0.0715
	lactate	1.04	0.6144	0.92	0.2998
	glycerate	1.44	0.1548	3.33	0.2178
Pentose Phosphate Pathway	6-phosphogluconate	1.03	0.8591	0.52	0.1415
	ribose 1-phosphate	1.16	0.8625	1.54	0.0758
	sedoheptulose-7-phosphate	1.08	0.9502	0.67	0.0713
Pentose Metabolism	ribose	1.37	0.7425	0.56	0.1232
	ribitol	0.83	0.3887	4.17	0.0000
	ribonate	0.79	0.2768	1.05	0.7446
	arabitol/xylitol	0.88	0.6180	0.85	0.0949
	ribulose/xylulose	1.13	0.9826	0.72	0.0610
	arabonate/xylonate	0.65	0.0584	0.88	0.7703
Glycogen Metabolism	maltotriose	1.32	0.5792	0.82	0.3739
	maltose	0.77	0.3147	0.86	0.2340
Disaccharides and Oligosaccharides	sucrose	0.92	0.1501	0.72	0.0781
Fructose, Mannose and Galactose Metabolism	fructose	1.23	0.7977	5.26	0.0018
	mannitol/sorbitol	1.67	0.0001	5.26	0.0000
	mannose	0.73	0.1755	1.59	0.2098
	galactitol (dulcitol)	1.59	0.0049	5.88	0.0000
	galactonate	1.12	0.5939	0.52	0.0459
Nucleotide Sugar	UDP-glucose	1.02	0.9603	0.79	0.0435
	UDP-galactose	0.98	0.8156	0.95	0.5945
	UDP-glucuronate	0.86	0.2832	2.86	0.0008
	guanosine 5'-diphospho-fucose	0.95	0.3243	0.88	0.2049
	UDP-N-acetylglucosamine	0.91	0.5493	1.85	0.0176
	cytidine 5'-monophospho-N-acetylneuraminic acid	0.95	0.5173	1.02	0.7891
	glucuronate 1-phosphate*	1.02	0.9330	1.12	0.9141
Aminosugar Metabolism	glucosamine-6-phosphate	0.59	0.2035	0.93	0.5260
	glucuronate	1.37	0.1144	0.55	0.0601
	N-acetylglucosamine 6-phosphate	0.44	0.0001	1.85	0.0218
	N-acetyl-glucosamine 1-phosphate	0.62	0.0059	1.92	0.0298
	N-acetylneuraminate	1.33	0.0091	1.28	0.0355
	N-acetylglucosaminylasparagine	1.04	0.7356	1.35	0.0510
	erythronate	0.62	0.0006	0.78	0.0862
	N-acetylglucosamine/N-acetylgalactosamine	0.48	0.0170	1.16	0.5073
	acetyl CoA	1.05	0.8623	0.49	0.0208

TCA Cycle	citrate	1.57	0.1553	0.81	0.6703
	aconitate [cis or trans]	1.34	0.1265	0.81	0.4944
	isocitrate	1.00	---	0.77	0.9400
	alpha-ketoglutarate	1.46	0.1885	1.00	0.9507
	succinylcarnitine	1.00	---	1.69	0.1163
	succinate	**2.35**	0.0054	2.00	0.1754
	fumarate	0.94	0.5320	1.15	0.3399
	malate	1.14	0.1746	1.00	0.9302
	itaconate	**1.25**	0.0044	0.96	0.5214
	2-methylcitrate/homocitrate	0.96	0.5622	0.92	0.3386
Oxidative Phosphorylation	acetylphosphate	1.58	0.0779	0.72	0.4221
	phosphate	1.05	0.8607	1.39	0.2977
Glycine, Serine and Threonine Metabolism	glycine	1.00	0.9445	1.02	0.7288
	N-acetylglycine	0.94	0.5414	**0.67**	0.0009
	betaine	**1.67**	0.0059	1.02	0.8111
	betaine aldehyde	**3.58**	0.0176	0.79	0.5341
	serine	**0.84**	0.0362	0.92	0.3883
	N-acetylserine	0.83	0.0671	**0.69**	0.0109
	threonine	**0.82**	0.0263	**0.79**	0.0155
	N-acetylthreonine	**0.78**	0.0020	0.77	0.0857
Alanine and Aspartate Metabolism	alanine	1.03	0.7824	0.92	0.3238
	N-acetylalanine	**0.74**	0.0002	**0.72**	0.0019
	aspartate	0.91	0.2782	**1.54**	0.0006
	asparagine	**0.84**	0.0215	0.81	0.0994
	N-acetylasparagine	**0.54**	0.0245	**0.66**	0.0089
	N-acetylaspartate (NAA)	**0.72**	0.0013	1.06	0.6514
Glutamate Metabolism	glutamate	1.07	0.1633	0.95	0.3746
	glutamine	**0.78**	0.0060	**0.79**	0.0365
	N-acetylglutamate	**0.58**	0.0006	**0.54**	0.0000
	N-acetylglutamine	0.99	0.8643	**0.67**	0.0332
	N-acetyl-aspartyl-glutamate (NAAG)	**0.57**	0.0001	**0.63**	0.0041
	gamma-aminobutyrate (GABA)	**0.57**	0.0238	1.12	0.7391
	4-hydroxyglutamate	**0.74**	0.0279	0.83	0.3725
	glutamate, gamma-methyl ester	0.55	0.0569	1.32	0.9520
	pyroglutamine	**0.62**	0.0003	1.02	0.9604
	gamma-carboxyglutamate	0.64	0.0716	0.93	0.9445
	S-1-pyrroline-5-carboxylate	1.34	0.1548	0.85	0.2448
Histidine Metabolism	histidine	0.90	0.1227	0.84	0.0864
	N-acetylhistidine	0.99	0.7980	**0.65**	0.0180
	1-methylhistidine	1.08	0.7985	**0.25**	0.0412
	imidazole propionate	0.59	0.0767	**0.62**	0.0067
	imidazole lactate	0.88	0.0623	**0.75**	0.0331
	4-imidazoleacetate	0.83	0.3523	**0.78**	0.0206
	histidine methyl ester	1.05	0.7255	**0.75**	0.0320
Lysine Metabolism	lysine	0.99	0.9633	0.91	0.4364
	N2-acetyllysine	0.90	0.3769	0.85	0.3739
	N6-acetyllysine	0.81	0.4627	0.83	0.3655
	N6,N6,N6-trimethyllysine	**0.75**	0.0038	**0.70**	0.0062
	5-hydroxylysine	0.87	0.6288	0.70	0.3356
	saccharopine	1.05	0.6735	**0.51**	0.0032

Lysine Metabolism	2-aminoadipate	0.73	0.0066	0.93	0.4462
	glutarate (pentanedioate)	0.92	0.4001	0.85	0.5083
	3-hydroxyglutarate	1.34	0.0516	0.75	0.0522
	pipecolate	0.89	0.5381	1.16	0.4593
	cadaverine	1.00	---	0.70	0.0159
	N-acetyl-cadaverine	1.00	---	1.18	0.5330
Phenylalanine and Tyrosine Metabolism	phenylalanine	1.17	0.0453	0.88	0.1220
	N-acetylphenylalanine	1.32	0.0660	0.56	0.0073
	phenyllactate (PLA)	2.13	0.0003	0.55	0.0095
	tyrosine	0.87	0.0315	0.88	0.1057
	N-acetyltyrosine	1.04	0.6709	1.00	---
	4-hydroxyphenylpyruvate	1.11	0.9800	0.81	0.3378
	3-(4-hydroxyphenyl)lactate	0.94	0.4205	0.61	0.0001
	phenol sulfate	1.38	0.5637	0.71	0.4741
	p-cresol sulfate	1.04	0.5997	2.17	0.0015
Tryptophan Metabolism	tryptophan	0.88	0.1448	0.85	0.0907
	N-acetyltryptophan	1.21	0.1632	1.00	---
	indolelactate	1.19	0.3739	0.76	0.2930
	kynurenine	1.07	0.6739	0.19	0.0027
	kynurenate	1.22	0.4634	1.10	0.3739
	C-glycosyltryptophan	1.00	---	0.39	0.0018
	N-acetylkynurenine (2)	1.09	0.5681	1.00	---
	thioproline	1.08	0.4787	1.02	0.7838
Leucine, Isoleucine and Valine Metabolism	leucine	0.98	0.5807	0.90	0.1857
	N-acetylleucine	1.06	0.5742	0.60	0.0018
	4-methyl-2-oxopentanoate	1.44	0.2516	0.71	0.3998
	isovalerylcarnitine	1.21	0.0728	0.58	0.1317
	beta-hydroxyisovalerate	1.04	0.6360	1.02	0.8807
	alpha-hydroxyisovalerate	1.79	0.0077	0.65	0.0209
	methylsuccinate	1.21	0.3019	0.94	0.5164
	isoleucine	0.99	0.7972	0.89	0.1264
	N-acetylisoleucine	1.07	0.6478	0.56	0.0214
	3-methyl-2-oxobutyrate	1.96	0.2298	0.83	0.5680
	3-methyl-2-oxovalerate	1.00	---	1.49	0.3739
	2-methylbutyrylcarnitine (C5)	1.12	0.1685	0.78	0.1901
	tiglylcarnitine	0.76	0.4733	0.75	0.6411
	2-hydroxy-3-methylvalerate	1.00	---	1.43	0.1295
	ethylmalonate	0.76	0.0246	1.39	0.0478
	valine	0.99	0.9248	0.90	0.2296
	N-acetylvaline	0.92	0.3326	0.68	0.0014
	isobutyrylcarnitine	1.24	0.2096	0.70	0.0568
	isobutyrylglycine	0.91	0.8019	0.94	0.7513
	3-hydroxyisobutyrate	1.79	0.2055	1.04	0.6944
	alpha-hydroxyisocaproate	1.65	0.1243	1.11	0.6292
	methionine	0.89	0.0675	0.84	0.1072
	N-acetylmethionine	0.77	0.0064	0.81	0.0534
	N-formylmethionine	0.64	0.0354	0.76	0.2808
	methionine sulfone	0.42	0.0228	0.29	0.0009
	methionine sulfoxide	1.12	0.2792	0.77	0.0373
	N-acetylmethionine sulfoxide	0.99	0.8626	1.00	0.9280

Methionine, Cysteine, SAM and Taurine Metabolism	S-adenosylmethionine (SAM)	1.04	0.9141	1.10	0.4577
	S-adenosylhomocysteine (SAH)	1.40	0.0919	0.68	0.0099
	cystathionine	1.29	0.0192	0.49	0.0007
	cysteine	1.06	0.5899	1.32	0.1382
	N-acetylcysteine	1.03	0.8382	0.89	0.3314
	cystine	3.80	0.0728	0.81	0.2719
	S-methylcysteine	1.32	0.1557	1.69	0.0741
	cysteine s-sulfate	1.47	0.5426	0.89	0.4738
	cysteine sulfinic acid	0.11	0.0001	1.39	0.1550
	hypotaurine	0.77	0.0224	0.78	0.2925
	taurine	0.98	0.9662	0.71	0.1840
	N-acetyltaurine	0.64	0.0441	0.41	0.0778
	2-hydroxybutyrate/2-hydroxyisobutyrate	0.98	0.9402	1.11	0.4063
Urea cycle; Arginine and Proline Metabolism	arginine	1.26	0.5509	0.91	0.4739
	ornithine	1.21	0.2189	0.61	0.0191
	proline	0.91	0.1903	0.65	0.0003
	citrulline	1.07	0.7916	0.61	0.2270
	argininosuccinate	0.39	0.1833	1.04	0.6581
	homoarginine	1.10	0.9725	0.61	0.0435
	homocitrulline	0.98	0.9095	0.38	0.0064
	dimethylarginine (SDMA + ADMA)	0.87	0.1064	0.76	0.2147
	N-acetylarginine	0.92	0.7838	1.00	---
	N-delta-acetylornithine	0.95	0.6637	0.50	0.0008
	trans-4-hydroxyproline	0.72	0.0302	1.08	0.4013
	pro-hydroxy-pro	0.67	0.1217	0.34	0.0447
	N-monomethylarginine	1.09	0.4497	1.11	0.7973
	N-acetylcitrulline	1.02	0.3739	1.00	---
Creatine Metabolism	creatine	0.62	0.0001	1.30	0.1063
	creatinine	0.77	0.0047	1.39	0.0166
	creatine phosphate	0.26	0.0300	1.37	0.2024
	guanidinoacetate	4.30	0.0001	0.76	0.0270
Polyamine Metabolism	putrescine	0.99	0.9502	0.47	0.0021
	spermidine	0.61	0.0081	0.56	0.0383
	5-methylthioadenosine (MTA)	1.15	0.2972	1.05	0.5285
	N-acetylputrescine	1.22	0.0893	0.88	0.6826
Guanidino and Acetamido Metabolism	4-guanidinobutanoate	0.72	0.0074	1.10	0.4608
Glutathione Metabolism	glutathione, reduced (GSH)	0.44	0.1780	16.67	0.1415
	glutathione, oxidized (GSSG)	0.78	0.3628	12.50	0.1778
	cysteine-glutathione disulfide	1.24	0.8266	4.76	0.1107
	S-methylglutathione	0.86	0.5820	1.22	0.1813
	cysteinylglycine	0.87	0.4253	2.70	0.3124
	5-oxoproline	1.17	0.2661	0.94	0.5553
	ophthalmate	1.56	0.6992	4.17	0.1780
Glycerolipid Metabolism	glycerol	1.33	0.2758	1.30	0.1716
	glycerol 3-phosphate	1.06	0.7495	1.69	0.0265
	glycerophosphoglycerol	1.15	0.1952	1.03	0.8155

List of detected metabolites. Statistical comparison of fold-changes between groups (TβT-PLC/PLC, SNUshTβRI/SNUsh-) was performed by Welch's two-sample t-test. p <0.05 was considered significant. Values <1 indicate downregulation (green shading colour when significant) and values >1 indicate upregulation (red shading colour when significant) as compared to respective control.

Supplementary Table S4

List of analysed genes in PLC vs TβT-PLC and in SNU449shTβRI vs SNU449sh-

Sub pathway	Symbol	GenBank	Description	TβT-PLC PLC	p-value	SNU449sh-SNU449shTβRI	p-value
Glycolysis	***ALDOA***	NM_000034	Aldolase A, fructose-bisphosphate	0.72	0.6632	1.24	0.4730
	ALDOB	NM_000035	Aldolase B, fructose-bisphosphate	N/A	N/A	N/A	N/A
	ALDOC	NM_005165	Aldolase C, fructose-bisphosphate	N/A	N/A	N/A	N/A
	BPGM	NM_001724	2,3-bisphosphoglycerate mutase	1.05	0.8906	**2.30**	0.0378
	ENO1	NM_001428	Enolase 1, (alpha)	0.64	0.5165	1.27	0.5646
	ENO2	NM_001975	Enolase 2 (gamma, neuronal)	1.90	0.0930	2.23	0.0869
	ENO3	NM_001976	Enolase 3 (beta, muscle)	**1.76**	0.0045	2.44	0.3031
	GALM	NM_138801	Galactose mutarotase (aldose 1-epimerase)	0.78	0.2395	1.91	0.1528
	GCK	NM_000162	Glucokinase (hexokinase 4)	N/A	N/A	N/A	N/A
	GPI	NM_000175	Glucose-6-phosphate isomerase	1.24	0.6794	1.04	0.8704
	HK2	NM_000189	Hexokinase 2	0.54	0.1928	N/A	N/A
	HK3	NM_002115	Hexokinase 3 (white cell)	N/A	N/A	N/A	N/A
	LDHA	NM_005566	Lactate dehydrogenase A	**1.57**	0.0042	1.18	0.3012
	LDHB	NM_002300	Lactate dehydrogenase B	**0.05**	0.0009	1.47	0.1361
	PFKL	NM_002626	Phosphofructokinase, liver	0.60	0.4618	1.45	0.1226
	PGAM2	NM_000290	Phosphoglycerate mutase 2 (muscle)	1.58	0.4283	2.40	0.1873
	PGK1	NM_000291	Phosphoglycerate kinase 1	0.83	0.3954	1.50	0.3190
	PGK2	NM_138733	Phosphoglycerate kinase 2	0.67	0.4785	1.47	0.6054
	PGM1	NM_002633	Phosphoglucomutase 1	0.75	0.5668	**1.69**	0.0382
	PGM2	NM_018290	Phosphoglucomutase 2	1.24	0.3605	1.50	0.1576
	PGM3	NM_015599	Phosphoglucomutase 3	0.93	0.4585	1.28	0.2764
	PKLR	NM_000298	Pyruvate kinase, liver and RBC	0.54	0.2192	N/A	N/A
	PKM2	NM_002654	Pyruvate kinase, muscle 2	0.71	0.1108	1.24	0.0783
	TPI1	NM_000365	Triosephosphate isomerase 1	1.00	0.9960	1.24	0.2339
Pyruvate dehydrogenase regulation	***PDK1***	NM_002610	Pyruvate dehydrogenase kinase, isozyme 1	0.94	0.7184	2.05	0.1985
	PDK2	NM_002611	Pyruvate dehydrogenase kinase, isozyme 2	1.08	0.8779	**1.53**	0.0369
	PDK3	NM_005391	Pyruvate dehydrogenase kinase, isozyme 3	1.00	0.9913	1.02	0.9198
	PDK4	NM_002612	Pyruvate dehydrogenase kinase, isozyme 4	0.98	0.9599	**2.81**	0.0058
	PDP2	NM_020786	Pyruvate dehyrogenase phosphatase catalytic subunit 2	0.77	0.4031	1.58	0.2832
	PDPR	NM_017990	Pyruvate dehydrogenase phosphatase regulatory subunit	0.91	0.8375	1.34	0.1627
Pyruvate dehydrogenase complex	***DLAT***	NM_001931	Dihydrolipoamide S-acetyltransferase	0.69	0.3955	1.13	0.6535
	DLD	NM_000108	Dihydrolipoamide dehydrogenase	1.47	0.2840	1.48	0.1506
	DLST	NM_001933	Dihydrolipoamide S-succinyltransferase	0.73	0.5973	1.02	0.9312
	PDHA1	NM_000284	Pyruvate dehydrogenase (lipoamide) alpha 1	0.88	0.4449	1.19	0.1892
	PDHB	NM_000925	Pyruvate dehydrogenase (lipoamide) beta	0.95	0.8239	1.25	0.0768
	FBP1	NM_000507	Fructose-1,6-bisphosphatase 1	N/A	N/A	N/A	N/A
	FBP2	NM_003837	Fructose-1,6-bisphosphatase 2	0.92	0.7602	**2.02**	0.0358

Gluconeogenesis	***G6PC***	NM_000151	Glucose-6-phosphatase, catalytic subunit	**6.09**	0.0281	N/A	N/A
	G6PC3	NM_138387	Glucose 6 phosphatase, catalytic, 3	0.92	0.8920	1.30	0.1352
	PC	NM_000920	Pyruvate carboxylase	0.90	0.8322	1.65	0.3769
	PCK1	NM_002591	Phosphoenolpyruvate carboxykinase 1 (soluble)	N/A	N/A	N/A	N/A
	PCK2	NM_004563	Phosphoenolpyruvate carboxykinase 2	0.52	0.2829	1.11	0.7792
Pentose Phosphate Pathway	***G6PD***	NM_000402	Glucose-6-phosphate dehydrogenase	0.51	0.2774	**1.79**	0.0067
	H6PD	NM_004285	Hexose-6-phosphate dehydrogenase (glucose 1-dehydrogenase)	0.81	0.7285	**2.37**	0.0051
	PGLS	NM_012088	6-phosphogluconolactonase	0.80	0.6103	**1.29**	0.0457
	PRPS1	NM_002764	Phosphoribosyl pyrophosphate synthetase 1	0.74	0.5171	1.06	0.8340
	PRPS1L1	NM_175886	Phosphoribosyl pyrophosphate synthetase 1-like 1	N/A	N/A	N/A	N/A
	PRPS2	NM_002765	Phosphoribosyl pyrophosphate synthetase 2	1.20	0.6137	0.99	0.9821
	RBKS	NM_022128	Ribokinase	1.52	0.1987	0.70	0.1863
	RPE	NM_199229	Ribulose-5-phosphate-3-epimerase	1.38	0.4522	1.14	0.6628
	RPIA	NM_144563	Ribose 5-phosphate isomerase A	0.98	0.9452	1.11	0.6956
	TALDO1	NM_006755	Transaldolase 1	0.92	0.8431	1.05	0.8629
	TKT	NM_001064	Transketolase	0.91	0.8592	1.00	0.9883
Metabolism of glycogen	***AGL***	NM_000028	Amylo-alpha-1, 6-glucosidase, 4-alpha-glucanotransferase	1.33	0.3256	1.01	0.9619
	GBE1	NM_000158	Glucan (1,4-alpha-), branching enzyme 1	1.72	0.2010	1.17	0.4905
	GSK3A	NM_019884	Glycogen synthase kinase 3 alpha	0.82	0.7598	1.28	0.4429
	GSK3B	NM_002093	Glycogen synthase kinase 3 beta	0.89	0.6583	1.95	0.0691
	GYS1	NM_002103	Glycogen synthase 1 (muscle)	0.54	0.3406	2.06	0.2185
	GYS2	NM_021957	Glycogen synthase 2 (liver)	N/A	N/A	N/A	N/A
	PGM1	NM_002633	Phosphoglucomutase 1	0.75	0.5668	**1.69**	0.0382
	PGM2	NM_018290	Phosphoglucomutase 2	1.24	0.3605	1.50	0.1576
	PGM3	NM_015599	Phosphoglucomutase 3	0.93	0.4585	1.28	0.2764
	PHKA1	NM_002637	Phosphorylase kinase, alpha 1 (muscle)	1.02	0.9702	1.37	0.2345
	PHKB	NM_000293	Phosphorylase kinase, beta	1.65	0.2256	0.98	0.9146
	PHKG1	NM_006213	Phosphorylase kinase, gamma 1 (muscle)	0.52	0.5657	2.18	0.1770
	PHKG2	NM_000294	Phosphorylase kinase, gamma 2 (testis)	N/A	N/A	**1.46**	0.0488
	PYGL	NM_002863	Phosphorylase, glycogen, liver	0.9650	0.9200	1.05	0.5472
	PYGM	NM_005609	Phosphorylase, glycogen, liver	N/A	N/A	N/A	N/A
	UGP2	NM_006759	UDP-glucose pyrophosphorylase 2	1.55	0.1088	1.27	0.1431
	ACLY	NM_001096	ATP citrate lyase	0.62	0.3959	1.55	0.1602
	ACO1	NM_002197	Aconitase 1, soluble	0.89	0.7232	**1.52**	0.0174
	ACO2	NM_001098	Aconitase 2, mitochondrial	0.74	0.5841	1.07	0.7967
	CS	NM_004077	Citrate synthase	0.82	0.7312	1.26	0.1625
	FH	NM_000143	Fumarate hydratase	0.87	0.4614	1.31	0.2478
	IDH1	NM_005896	Isocitrate dehydrogenase 1 (NADP+), soluble	0.90	0.7141	1.55	0.1507

	IDH2	NM_002168	Isocitrate dehydrogenase 2 (NADP+), mitochondrial	0.88	0.7952	1.24	0.4041
	IDH3A	NM_005530	Isocitrate dehydrogenase 3 (NAD+) alpha	0.88	0.6725	1.18	0.6149
	IDH3B	NM_174856	Isocitrate dehydrogenase 3 (NAD+) beta	0.94	0.9032	1.01	0.9706
	IDH3G	NM_174869	Isocitrate dehydrogenase 3 (NAD+) gamma	0.69	0.5370	1.30	0.3292
	MDH1	NM_005917	Malate dehydrogenase 1, NAD (soluble)	1.06	0.7400	**1.38**	0.0219
	MDH1B	NM_00103984	Malate dehydrogenase 1B, NAD (soluble)	N/A	N/A	N/A	N/A
	MDH2	NM_005918	Malate dehydrogenase 2, NAD (mitochondrial)	0.91	0.8429	**1.57**	0.0465
**TCA cycle**	***OGDH***	NM_002541	Oxoglutarate dehydrogenase	0.83	0.7126	**2.06**	0.0402
	PC	NM_000920	Pyruvate carboxylase	0.90	0.8322	1.65	0.3769
	PCK1	NM_002591	Phosphoenolpyruvate carboxykinase 1 (soluble)	N/A	N/A	N/A	N/A
	PCK2	NM_004563	Phosphoenolpyruvate carboxykinase 2	0.52	0.2829	1.11	0.7792
	SDHA	NM_004168	Succinate dehydrogenase complex, subunit A, flavoprotein (Fp)	0.83	0.7063	1.04	0.7809
	SDHB	NM_003000	Succinate dehydrogenase complex, subunit B, iron sulfur (Ip)	1.05	0.8189	1.08	0.7340
	SDHC	NM_003001	Succinate dehydrogenase complex, subunit C, integral membrane protein	1.61	0.3011	1.32	0.2450
	SDHD	NM_003001	Succinate dehydrogenase complex, subunit D, integral membrane protein	1.65	0.1828	1.32	0.1972
	SUCLA2	NM_003850	Succinate-CoA ligase, ADP-forming, beta subunit	2.56	0.1072	1.05	0.8464
	SUCLG1	NM_003849	Succinate-CoA ligase, alpha subunit	0.91	0.2485	1.30	0.0788
	SUCLG2	NM_003848	Succinate-CoA ligase, GDP-forming, beta subunit	1.74	0.2929	1.16	0.6245
	GLS1	NM_014905	Glutaminase 1	**1.55**	0.0248	**1.30**	0.0229
	GLS2	NM_013267	Glutaminase 2	N/A	N/A	N/A	N/A
**Glutamine Metabolism**	***SLC1A5***	NM_005628	Solute carrier family 1 (neutral amino acid transporter), member 5	0.72	0.2682	1.24	0.3093
	SLC7A5	NM_003486	Solute carrier family 7 (amino acid transporter light chain, L system), member 5	1.35	0.3617	**1.63**	0.0169

List of analysed genes. Expression in fold comparing TβT-PLC/PLC and SNU449sh-/SNU449shTβRI. $p<0.05$ was considered significant. Values <1 indicate downregulation (green shading colour when significant) and values >1 indicate upregulation (red shading colour when significant) as compared to respective control. N/A indicates Ct values >35 and considered as non-detectable gene expression.

Supplementary Table S5. Molecular characteristics of the human HCC cell lines used in this study					
Cell line	Tumor Type	Differentiated grade	*TP53* status	Other Characteristics	TGF-β Signature
PLC/PRF/5	Human Liver Hepatocarcinoma	Well-differentiated	Mutation R249S	Express *ABL1*, *FES*, *MYC*, *HRAS*, and *PDGFB* oncogenes	Early
Huh7	Human Liver Hepatocarcinoma	Well-differentiated	Mutation Y220C		Early
Hep3B	Human Negroid Hepatocarcinoma	Well-differentiated	Deleted	Deficient in functional RB1; mutations within *FAS*	Early
SNU449	Human Asian Hepatocarcinoma	Poorly-differentiated	Mutations A161T K138R	Mutation in *CDKN2A*	Late
HLE	Human Liver Hepatocarcinoma	Poorly-differentiated	Mutation G244A R249S V272M	-	Late

Supplementary Table S6. List of antibodies used in this study

Primary antibody	Secondary antibody	Company	Reference code	Application
E-CADHERIN	Anti-mouse	BD Biosciences	BD-610182	IF
VIMENTIN	Anti-mouse	Sigma-Aldrich	V6630	IF
F-ACTIN	TRITC-conjugated	Sigma-Aldrich	P1951	IF
Ki67	Anti-rabbit	Abcam	ab16667	IF
TGFβRI	Anti-rabbit	Santa Cruz Biotechnology	sc-399	WB, FC
β-ACTIN	Anti-mouse	Sigma-Aldrich	A5441	WB
**Secondary antibody**	**Company**	**Reference code**	**Application**	
Alexa 488 goat anti mouse	Life Technologies	A11001	IF	
Alexa 488 goat anti rabbit	Life Technologies	A11008	IF	
Anti-mouse	GE Healthcare	NA931V	WB	
Anti-rabbit	GE Healthcare	NA934V	WB, FC	

IF: Immunofluorescence; WB: Western Blot; FC; Flow Cytometry
